# Supplementary material for: An ALE meta-analytic review of top-down and bottom-up processing of music in the brain
Source: Sci Rep. 2021 Oct 21;11:20813. doi: 10.1038/s41598-021-00139-3 (PMC8531391; doi:10.1038/s41598-021-00139-3)
Supplement: Supplementary file 1 — Supplementary Information. [file 41598_2021_139_MOESM1_ESM.pdf]

SUPPLEMENTARY INFORMATION

An ALE meta-analytic review of top-down and bottom-up processing of music in the brain.

Pando-Naude, Victor[1+*], Patyczek, Agata[2+], Bonetti, Leonardo[1], Vuust, Peter[1]

[1]Center for Music in the Brain, Department of Clinical Medicine, Aarhus University & The Royal Academy of Music Aarhus/Aalborg, Denmark
[2]MR Center of Excellence, Center for Medical Physics and Biomedical Engineering, Medical University of Vienna, Austria

[+] Shared first authorship
*Corresponding Author

Table of Contents

Search strings. **2**

Supplementary Figure 1. PRISMA flowchart for literature search process. **3**

PRISMA Checklist **4**

Supplementary Table 1. Characteristics of MRI acquisition and analysis. **6**

Supplementary Table 2. MRI quality. **9**

Supplementary Table 3. Contrast analyses comparing music perception, production and imagery, at cluster level inference p < 0.05 (FWE) **12**

Supplementary Table 4. Meta-analytic connectivity modeling results from primary outcomes (n = 17 ROIs), at cluster level inference p < 0.05 (FWE) **14**

Supplementary Table 5. Functional characterization of brain regions resulted from primary outcomes (n = 17 ROIs) according to BrainMap database **22**

Supplementary Table 6. FSN robustness assessment for significant ALE maps of music perception, production, and imagery **26**

Citations of included studies **27**

Abbreviations **27**

References **28**

Search strings.

Last search date: 26.02.2021

PsycInfo

(music imagery OR music listening OR music production OR music composition OR music performance OR music playing OR music perception OR auditory imagery) AND magnetic resonance imaging

612 results

Scopus

(TITLE-ABS-KEY (music imagery) OR TITLE-ABS-KEY (music listening) OR TITLE-ABS-KEY (music production) OR TITLE-ABS-KEY (music performance) OR TITLE-ABS-KEY (music playing) OR TITLE-ABS-KEY (music composition) OR TITLE-ABS-KEY (music perception)) AND TITLE-ABS-KEY (magnetic resonance imaging)

963 results

PubMed

(“music imagery” [All Fields] OR “music listening” [All Fields] OR “music production” [All Fields] OR “music performance” [All Fields] OR “music playing” [All Fields] OR “music composition” [All Fields] OR “music perception” [All Fields]) AND (“magnetic resonance imaging” [MeSH Terms] OR “magnetic resonance imaging” [All Fields])

132 results

Supplementary Figure 1. PRISMA flowchart for literature search process.

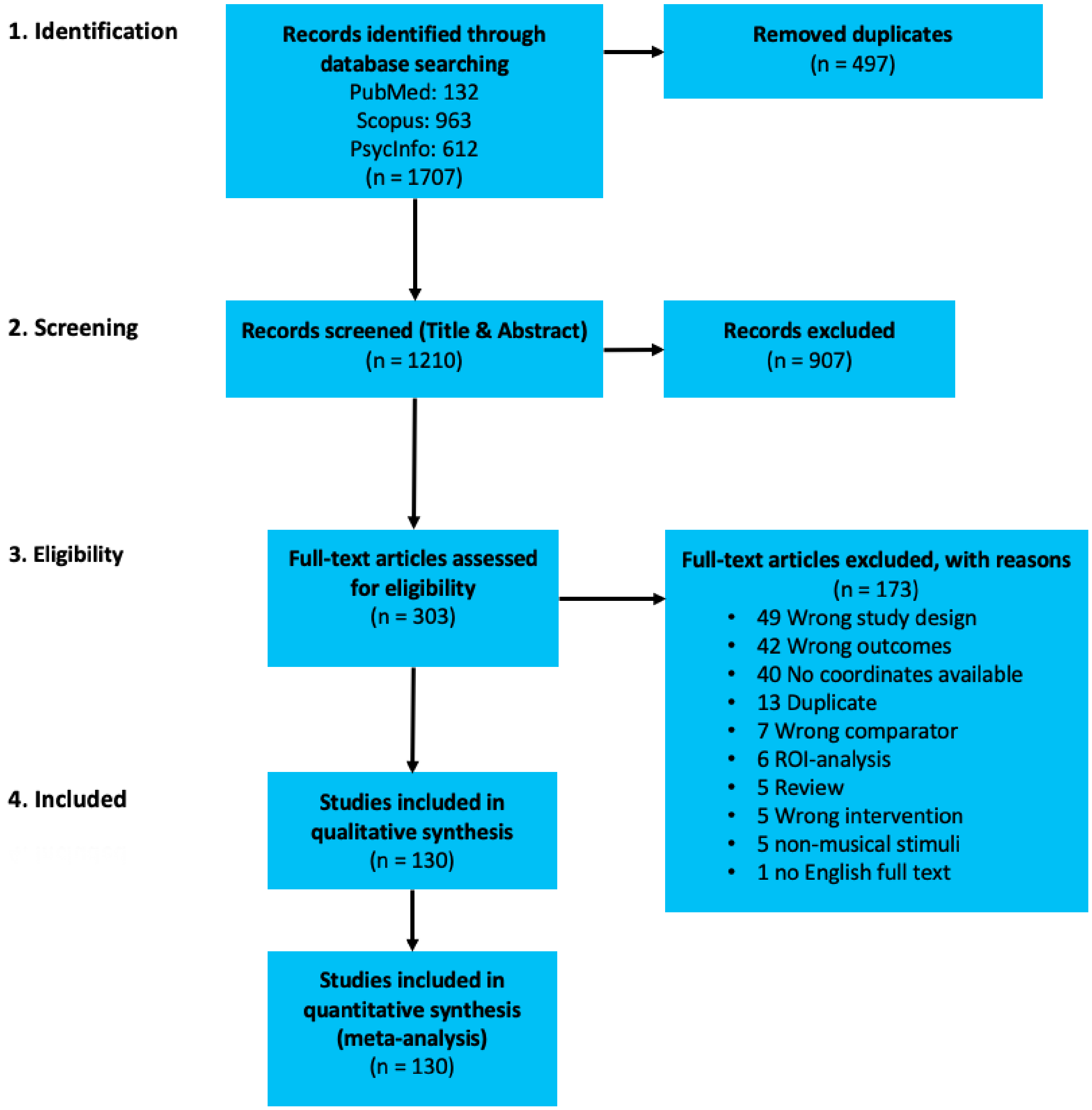

Supplementary Figure 1. PRISMA flowchart for literature search process.

PRISMA Checklist

SI = Supplementary Information

Section/topic	#	Checklist item	Reported on page #
**TITLE**			
Title	1	Identify the report as a systematic review, meta-analysis, or both.	1
**ABSTRACT**			
Structured summary	2	Provide a structured summary including, as applicable: background; objectives; data sources; study eligibility criteria, participants, and interventions; study appraisal and synthesis methods; results; limitations; conclusions and implications of key findings; systematic review registration number.	2
**INTRODUCTION**			
Rationale	3	Describe the rationale for the review in the context of what is already known.	3
Objectives	4	Provide an explicit statement of questions being addressed with reference to participants, interventions, comparisons, outcomes, and study design (PICOS).	4
**METHODS**			
Protocol and registration	5	Indicate if a review protocol exists, if and where it can be accessed (e.g., Web address), and, if available, provide registration information including registration number.	17
Eligibility criteria	6	Specify study characteristics (e.g., PICOS, length of follow-up) and report characteristics (e.g., years considered, language, publication status) used as criteria for eligibility, giving rationale.	17
Information sources	7	Describe all information sources (e.g., databases with dates of coverage, contact with study authors to identify additional studies) in the search and date last searched.	17
Search	8	Present full electronic search strategy for at least one database, including any limits used, such that it could be repeated.	SI, p.2
Study selection	9	State the process for selecting studies (i.e., screening, eligibility, included in systematic review, and, if applicable, included in the meta-analysis).	17
Data collection process	10	Describe method of data extraction from reports (e.g., piloted forms, independently, in duplicate) and any processes for obtaining and confirming data from investigators.	17
Data items	11	List and define all variables for which data were sought (e.g., PICOS, funding sources) and any assumptions and simplifications made.	17
Risk of bias in individual studies	12	Describe methods used for assessing risk of bias of individual studies (including specification of whether this was done at the study or outcome level), and how this information is to be used in any data synthesis.	17
Summary measures	13	State the principal summary measures (e.g., risk ratio, difference in means).	18
Synthesis of results	14	Describe the methods of handling data and combining results of studies, if done, including measures of consistency (e.g., I^2) for each meta-analysis.	18

Section/topic	#	Checklist item	Reported on page #
Risk of bias across studies	15	Specify any assessment of risk of bias that may affect the cumulative evidence (e.g., publication bias, selective reporting within studies).	18
Additional analyses	16	Describe methods of additional analyses (e.g., sensitivity or subgroup analyses, meta-regression), if done, indicating which were pre-specified.	18
**RESULTS**			
Study selection	17	Give numbers of studies screened, assessed for eligibility, and included in the review, with reasons for exclusions at each stage, ideally with a flow diagram.	5
Study characteristics	18	For each study, present characteristics for which data were extracted (e.g., study size, PICOS, follow-up period) and provide the citations.	5
Risk of bias within studies	19	Present data on risk of bias of each study and, if available, any outcome level assessment (see item 12).	5
Results of individual studies	20	For all outcomes considered (benefits or harms), present, for each study: (a) simple summary data for each intervention group (b) effect estimates and confidence intervals, ideally with a forest plot.	6
Synthesis of results	21	Present results of each meta-analysis done, including confidence intervals and measures of consistency.	6-10
Risk of bias across studies	22	Present results of any assessment of risk of bias across studies (see Item 15).	SI p.26
Additional analysis	23	Give results of additional analyses, if done (e.g., sensitivity or subgroup analyses, meta-regression [see Item 16]).	6-10
**DISCUSSION**			
Summary of evidence	24	Summarize the main findings including the strength of evidence for each main outcome; consider their relevance to key groups (e.g., healthcare providers, users, and policy makers).	11
Limitations	25	Discuss limitations at study and outcome level (e.g., risk of bias), and at review-level (e.g., incomplete retrieval of identified research, reporting bias).	14
Conclusions	26	Provide a general interpretation of the results in the context of other evidence, and implications for future research.	16
**FUNDING**			
Funding	27	Describe sources of funding for the systematic review and other support (e.g., supply of data); role of funders for the systematic review.	20

Supplementary Table 1. Characteristics of MRI acquisition and analysis.

			MRI acquisition				T1				T2				Analysis	
	Author	Year	Teslas	MRI-system	MRI-model	Head-coil	Sequence	TR (ms)	TE (ms)	Voxel size (mm)	Sequence	TR (ms)	TE (ms)	Voxel size (mm)	Software	Method
1	Agustus	2018	3	Siemens	Trio	12-channel	T1-w	-	-	-	EPI	11360	30	3x3x2	SMP8	MPM
2	Alluri	2012	3	GE	Signa	-	T1-w	-	-	-	EPI	2000	32	-	SPM8	GLM, PCA
3	Alluri	2013	3	GE	Signa	standard	T1-w	6.552	2.824	0.94x0.94x1.2	EPI	2200	30	-	SPM8	PCR
4	Alonso	2016	3	Siemens	Trio	-	MP2RAGE	2300	4.18	1x1x1	EPI	2100	29	3x3x3	SPM8	GLM
5	Alttenmüller	2014	3	Siemens	Allegra	8	T1-w	1550	7.3	1x1x1	T2-w	2000	30	-		GLM
6	Angulo-P	2014	3	GE	MR750	32	T1-w	2300	3	1x1x1	EPI	3000	40	2x2x3	FSL	GLM
7	Armony	2015	3	GE	MR750	32	T1-w	2300	3	2x2x3	EPI	3000	40	2x2x3	SPM8	LOSO
8	Bangert	2006	1.5	GE	Signa	quadrature	SPGR	-	-	-	EPI	4500	40	-	SPM99	GLM
9	Barrett	2016	3	Siemens	Trio	-	T1-w	2500	4.82	-	EPI	2000	25	1x1x1	SPM5	GLM
10	Barrett	2018	3	Phillips	Achieva	32	MPRAGE	-	-	0.7x0.7x0.7	EPI	2500	25	-	SPM12	Custom
11	Barrett	2020	3	Phillips	Achieva	32	MPRAGE	-	-	1x1x1	EPI	2000	30	-	SPM12	GLM
12	Bastepe-G	2020	1.5	Siemens	Aera	20	MPRAGE	1900	2.84	-	EPI	4000	50	-	SPM12	GLM
13	Baumann	2007	3	Phillips	Intera	body coil	T1-w	-	-	0.98x0.98x0.75	EPI	2000	35	2.75x2.74x4.5	Matlab	GLM
14	Bengtsson	2006	1.5	GE	Signa Horizon	-	T1-w	-	-	1x1x1	EPI	4000	60	-	SPM99	GLM
15	Bengtsson	2007	1.5	GE	Signa Horizon	-	T1-w	-	-	0.86x0.86x2	EPI	4000	60	-	SPM99	GLM
16	Bengtsson	2009	3	Siemens	Electra	-	T1-w	-	-	1x1x1	EPI	3000	40	-	SPM	-
17	Bianco	2016	3	Siemens	Trio	32	MP2RAGE	5000	2.03	1x1x1	EPI	2000	30	2.3x2.3x2.3	SPM8	GLM
18	Bishop	2013	3	Siemens	Magnetom	-	MPRGE	1830	4.43	-	EPI	3000	31	3x3x3	SPM2	-
19	Blood	1999	-	Siements	HR+	-	-	-	-	-	-	-	-	-	-	GLM
20	Bodner	2001	1.5	Siemens	Magnetom	birdcage	-	4000	4	-	EPI	3000	40	-	APM99	GLM
21	Bogert	2016	3	Siemens	Magnetom	20	T1-w	-	-	1x1x1	EPI	2000	32	3x3x4	SPM8	GLM
22	Brattico	2016	1.5	GE	Signa	-	T1-w	-	-	-	EPI	3000	32	-	SPM8	GLM
23	Bravo	2017a	3	GE	Signa	-	T1-w	-	-	1x1x1	EPI	3000	40	-	SPM8	PPI
24	Bravo	2017b	3	GE	Signa	-	T1-w	-	-	1x1x1	EPI	3000	40	-	SPM8	Regression
25	Bravo	2020	3	GE	Signa	-	T1-w	-	-	1x1x1	EPI	3000	40	-	SPM12	-
26	Brown	2004	-	CTI	HR+	-	-	-	-	-	-	-	-	-	-	-
27	Brown	2007	2	Elsinc	Gyrex	-	T1-w	-	-	1x1x1	EPI	2000	-	-	-	-
28	Chapin	2010	3	GE	Signa	-	SPGR	325	-	-	EPI	1200	35	3.75x3.75x4	AFNI	GLM
29	Chen	2008a	1.5	Siemens	Sonata	-	T1-w	-	-	1x1x1	EPI	1000	50	5x5x5	AFNI	GLM
30	Chen	2008b	1.5	Siemens	Sonata	-	T1-w	-	-	1x1x1	EPI	10000	50	5x5x5	AFNI	GLM
31	Chiang	2018	3	Siemens	Tim Trio	-	MPRAGE	1900	2.26	1x1x1	EPI	3000	35	3x3x3.5	FSL	GLM
32	Danielsen	2014	1.5	GE	Signa	-	FSPGR	-	-	-	EPI	3000	40	-	SPM8	GLM
33	Demorest	2009	1.5	GE	Signa	custom	FSPGR	-	-	-	EPI	3000	50	-	FSL	ICA
34	Donnay	2014	3	Philips	-	quadrature	-	-	-	-	EPI	2000	30	-	-	-
35	Engel	2011	3	Brucker	Medspeck	birdcage	MPRAGE	1300	-	1x1x1.5	EPI	1300	10	-	SPM5	GLM
36	Escoffier	2013	3	Siemens	Magentom	-	MPRAGE	2530	1.64	1x1x1	EPI	-	-	-	FSL	GLM
37	Fedorenko	2012	3	Siemens	Trio	32	T1-w	2000	2.39	1.33x1.33x1.33	EPI	2000	30	-	SPM5	GLM
38	Flores-G	2007	1.5	GE	v9.x	quadrature	T1-w	-	-	-	EPI	3000	60	4x4x8	SPM2	GLM
39	Fujisawa	2011		Magnex	Marconi						EPI	6000	55	3x3x3	SPM2	Box-car
40	González-G	2016	1.5	Phillips	Achieva	-	T1-w	10.2	4.2	1x1x1	EPI	1000	40	4x4x4	SPM8	GLM
41	Grahn	2007	3	Brucker	Medspec	-	SPGR	-	-	-	EPI	1100	37.5	-	SPM99	GLM
42	Grahn	2009a	3	Siemens	Trio	-	MRAGE	-	-	-	EPI	-	-	1x1x1	SPM5	GLM
43	Grahn	2009b	3	Siemens	Trio	-	MPRAGE	2250	2.99	1x1x1	EPI	2190	30	-	SPM5	PPI
44	Grahn	2013	3	Siemens	Trio	-	MPRAGE	2250	2.99	1x1x1	EPI	2190	30	-	SPM5	U
45	Green	2008	1.5	GE	Signa Excite	-	T1-w	-	-	-	EPI	2700	40	-	SPM5	U
46	Green	2012	1.5	GE	Signa	-	T1-w	-	-	-	EPI	2700	40	-	SPM5	-
47	Green	2018	3	Siemens	Magnetom	32	MPRAGE	-	-	-	EPI	2000	24	3.4x3.4x3	FSL	GLM
48	Halpern	1999	-	Siemens	Exact HR+	-	-	-	-	-	-	-	-	-	-	-
49	Halpern	2004	1.5	Siemens	Vision	-	-	-	-	1x1x1	EPI	1000		5x5x5	Matlab	U
50	Herdener	2014	-	-	-	-	-	-	-	-	-	-	-	-	BrainVoyager	GLM
51	Herholtz	2012	3	Siemens	Trio	32	T1-w	-	-	1x1x1	EPI	2100	30	3.5x3.5x3.5	FSL	GLM
52	Huang	2016	3	Siemens	Tim Trio	-	T1-w	2530	2.43	1x1x1	EPI	2200	30	-	SPM8	GLM
53	Janata	2002a	3	GE	Signa	-	T1-w	-	-	2.6x2.6x5	EPI	-	-	2.6x2.6x5	SPM99	GLM
54	Janata	2002b	1.5	GE	Horizon	birdcage	T1-w	650	6.6	-	EPI	2000	35	-	SPM99	GLM
55	Janata	2009	3	Siemens	Trio	-	MPRAGE	2000	4.82	1x1x1	EPI	2000	25	-	SPM5	GLM

56	Jeong	2011	3	GE	Signa	8	SPGR	-	-	-	EPI	3000	35	-	SPM8	GLM
57	Jungblut	2012	3	Siemens	Trio	-	-	-	-	-	EPI	2200	30	3.44x3.44x3.74	SPM8	GLM
58	Khalfa	2005	3	Brucker	Medspec	-	T1-w	-	-	1x0.75x1.22	EPI	-	-	-	SPM99	GLM
59	Kleber	2007	1.5	Siemens	Vision	-	MPRAGE	-	-	-	EPI	3000	40	-	SPM2	GLM
60	Kleider-O	2019	3	Siemens	Tim Trio	12	MPRAGE	-	-	1x1x1	EPI	2250	4.18	3.4x3.4x4	SPM8	TFCE
61	Koelsch	2003	3	Brucker	Medspec	-	T1-w	-	-	-	EPI	3000	-	-	LIPSIA	GLM
62	Koelsch	2014	3	Siemens	Magnetom	-	T1-w	-	-	1x1x1	EPI	2000	30	-	LIPSIA	GLM
63	Koelsch	2018	3	Siemens	Tim Trio	-	MPRAGE	-	-	1x1x1	EPI	2000	30	-	LIPSIA 2.1	GLM
64	Kornysheva	2010	3	Siemens	Trio	-	T1-w	-	-	-	EPI	2000	30	-	LIPISIA	GLM
65	Langheim	2002	1.5	GE	Signa	quadrature	-	3500	3.75	-	Multi-slice	2000	24	3.74x3.75x3.75	SPM96	GLM
66	Leaver	2009	3	Siemens	Trio	-	T1-w	-	-	1x1x1	EPI	1000	30	3x3x3	BrainVoyager	GLM
67	Lee	2011	3	Phillips	Achieva	-	MPRAGE	-	-	1x1x1	EPI	2000	35	3x3x3	SPM5	U
68	Lehne	2013	3	Siemens	Tim Trio	-	MPRAGE	-	-	1x1x1	EPI	2000	30	-	SPM8	GLM
69	Levitin	2005	3	GE	Signa	-	-	-	-	-	-	-	-	-	-	-
70	Levitin	2016	3	Siemens	Trio	12	MPRAGE	2100	2.4	-	EPI	3000	30	-	SPM12	RDA
71	Li	2019	3	Siemens	-	-	T1-w	2000	2.52		continuous	2000	26	-	SPM12	GLM
72	Limb	2006	3	GE	-	quadrature	T1-w	-	-	-	EPI	2000	30	-	SPM99	GLM
73	Limb	2008	3	GE	-	quadrature	-	-	-	-	EPI	2000	30	-	SPM99	GLM
74	Liu	2018	3	GE	Signa	8	MPRAGE	-	-	-	EPI	2000	30	-	SPM8	ICA
75	Matthews	2020	3	Siemens	Tim Trio	32	T1-w	2420	3.7	1x1x1	EPI	2000	-	2.35x2.53x2.50	SPM12	GLM
76	Meister	2004	3	Philips	Gyroscan	quadrature	-	-	-	-	EPI	3587	50	-	SPM99	LME
77	Merrill	2012	3	Siemenss	Trio	-	T1-w	1300	7.4	-	EPI	2500	30	3x3x3	SPM8	GLM
78	Mizuno	2007	1.5	GE	Signa	-	T1-w	-	-	-	EPI	2000	50	-	SPM99	GLM
79	Montag	2011	1.5	Siemens	Avanto	-	-	-	-	-	EPI	3200	40	-	SMP5	GLM
80	Morrison	2003	1.5	GE	-	-	-	-	-	-	EPI	2500	50	-	MEDx	3.4.1
81	Mueller	2015	3	Brucker	Medspec	birdcage	MPRAGE	-	-	1x1x1	ISSS	-	-	2.5x2.5x2.5	SPM8	GLM
82	Ohnishi	2001	1.5	Siemens	Magentom	-	-	-	-	-	-	3000	-	-	SPM99	GLM
83	Park	2013	3	Siemens	Magnetom	TIM	MPRAGE	2400	3.06	-	EPI	3000	30	-	BrainVoyager	GLM
84	Park	2014	3	Siemens	Magentom	-	MPRAGE	2400	3.06	-	EPI	3000	30	-	SPM8	GLM
85	Parsons	2005	-	GE	-	-	-	-	-	1x1x1	-	-	-	-	-	-
86	Pereira	2011	1.5	Phillips	Gyroscan	-	SPGR	-	-	1x1x1	EPI	3000	50	3.56x3.56x4.0	FSL	GLM
87	Peretz	2009	1.5	Siemens	Sonata	-	T1-1	-	-	1x1x1	EPI	1150	50	5x5x7	Matlab	GLM
88	Petrini	2011	3	Siemens	Tim Trio	-	MPRAGE	1900	2.52		EPI	2000	30	3x3x3	BrainVoyager	GLM
89	Pfordresher	2014	3	GE	Signa excite	8	-	-	-	-	EPI	2000	35	1x1x1	SPM5	Box car
90	Ragert	2014	3	Siemens	Tim Trio	birdcage	T1-w	-	-	1x1x1	EPI	2000	28	3x3x3	model	GLM
91	Reiterer	2008	1.5	Siemens	Vision	-	-	-	-	-	EPI	3000	40	-	SPM2	GLM
92	Rogalsky	2011	3	Siemens	Achieva	-	SPGR	2500	1.3	1x1x1	EPI	2000	40	1x1x1	AFNI	Regression
93	Sammler	2010	3	Siemens	Trio	-	MPRAGE	2300	4.8	1x1x1	EPI	2120	25	3x3x3	SPM5	GLM
94	Schmithorst	2005	3	Bruker	Medspec		T1-w	-	-	-	EPI	3000	38	-	SPM	ICA
95	Schön	2010	3	Bruker	Medspec	-	T1-w	-	-	-	EPI	2166	-	3.5	SPM2	GLM
96	Schwenzer	2011	1.5	Siemens	Magnetom	-	-	-	-	-	EPI	6000	17	3.6x3.6x4	SPM2	GLM
97	Shany	2019	3	GE	Signa	8-channel	SPGR	8.9	3.5	1x1x1	EPI	3000	35	3	BrainVoyager	GLM
98	Sikka	2015	3	Siemens	Magnetom	12-channel	MPRAGE	1760	2.2	1x1x1	EPI	10500	30	3.3	SPM8	GLM
99	Singer	2016	3	GE	Signa	8-channel	SPGR	8.9	3.5	1x1x1	EPI	3000	35	3	BrainVoyager	DCA
100	Skouras	2014	3	Siemens	Magnetom	12-channel	MPRAGE	-	-	1x1x1	EPI	2000	30	3	LIPSIA	GLM
101	Spada	2014	3	Philips	Achieva	-	T1-w	7.3	3.5	1x1x1	EPI	12000	30	-	SPM8	GLM
102	Tabei	2015	1.5	Siemens	Symphony	-	T1-w	2200	3.93	1x1x1	EPI	4000	50	3	SPM5	GLM
103	Tachibana	2010	3	Siemens	Trio	-	T1-w	2250	3.06	1x1x1	EPI	6000	30	3x3x5	SPM5	GLM
104	Taruffi	2017	3	Siemens	Trio	-	MPRAGE	-	-	1x1x1	EPI	2250	30	3	LIPSIA	ECM
105	Tervaniemi	2000	-	-	-	-	-	-	-	-	-	-	-	-	-	-
106	Tervaniemi	2006	3	Bruker	Medspec	-	T1-w	-	-	-	EPI	3000	30	-	LIPSIA	GLM
107	Thaut	2008	1.5	Siemens	Magnetom	quadrature	MPRAGE	11.4	4.4	1x1x1	EPI	3000	40	2.7x2.7x2.7	SPM99	Box-car
108	Tillmann	2003	1.5	GE	Signa	-	T1-w	650	6.6	0.937x0.937x5.0)	EPI	2000	35	3.75x3.75x5	SPM99	GLM
109	Tillmann	2006	3	Siemens	Trio	-	T1-w	1300	7.4	0.8x0.8x4	EPI	2000	30	3x3x4	LIPSIA	GLM
110	Toiviainen	2014	3	GE	-	standard	T1-w	6552	2.82	0.94x0.94x1.2	EPI	2200	30	3x3x3	SPM8	LASSO
111	Trost	2012	3	Siemens	Trio	-	MPRAGE	1900	2.32	0.9x0.9x0.9	EPI	3000	30	3.5	SPM5	GLM
112	Trost	2014	3	Siemens	Trio	12-channel	MPRAGE	1900	2.32	0.9x0.9x0.9	EPI	1980	27.3	3.2	SPM8	GLM
113	Tsai	2010	3	Bruker	-	-	T1-w	-	-	0.94x0.94x5	EPI	2000	30	3.75x3.75x5	SPM2	GLM
114	Tsai	2012	3	Bruker	-	-	T1-w	-	-	0.94x0.94x5	EPI	3000	30	3.75x3.75x5	SPM5	GLM
115	Tsai	2018	3	Siemens	Magnetom	20-channel	MPRAGE	-	-	0.9x0.9x0.9	EPI	2500	30	-	SPM12	GLM
116	Tsai	2019	3	Siemens	Magnetom	20-channel	MPRAGE	2000	2.3	0.93x0.93x0.93	EPI	2500	30	2.5	SPM12	GLM

117	Uhlig	2013	3	Siemens	Trio	Birdcage	-	-	-	-	EPI	2000	28	3x3x3	FSL	GLM
118	Villarreal	2013	3	GE	HDx	8-channel	SPGR-IR	13	6.1	-	EPI	2300	35	3.75x3.75x4	SPM5	GLM
119	Vuust	2011	1.5	GE	-	-	SPGR	30	-	-	EPI	3200	40	4	SPM2	Box-car
120	Wallmark	2018	3	Siemens	Trio	-	MPRAGE	1900	2.26	-	EPI	5000	34	-	FSL	GLM
121	Watanabe	2008	1.5	Hitachi	-	-	T1-w	-	-	-	EPI	14000	50	4x4x5	SPM2	LME
122	Whitehead	2018	-	-	-	-	-	-	-	-	multi-band	529	35	2x2x2	AFNI	GLM
123	Wilson	2010	3	GE	Signa XL	Birdcage	-	-	-	-	EPI	3000	40	1.88x1.88x1	SPM8	GLM
124	Yoo	2001	1.5	Siemens	Magnetom	Birdcage	T1-w	700	10	-	EPI	2500	50	-	SPM99	GLM+GRF
125	Zarate	2008	1.5	Siemens	Sonata	-	T1-w	-	-	1x1x1	T2*-w	10000	85	5x5x5	AFNI	GLM
126	Zarate	2010	3	Siemens	Trio	-	T1-w	-	-	1x1x1	T2*-w	60000	10300	3.5x3.5x3.5	AFNI	GLM
127	Zatorre	1994	1.5	Philips	Gyroscan	-	-	-	-	-	-	-	-	1.5x1.5.1.5	-	MSDCI
128	Zatorre	1996	1.5	Philips	Gyroscan	-	-	-	-	-	-	-	-	-	AFNI	RFT
129	Zatorre	2010	3	Siemens	Trio	8-channel	T1-w	-	-	1x1x1	EPI	2400		3.5x3.5x3.5	fMRISTAT	GLM
130	Zvyagintsev	2013	3	Siemens	Trio	12-channel	MPRAGE	2300	2.98	1x1x1	EPI	2000	28	3x3x3	BrainVoyager	GLM

GM, grey matter; WM, white matter; MRI, magnetic resonance imaging; FFE, fast field echo sequence; FLASH, fast low angle shot sequence; FSL, functional MRI of the brain software library; GE, gradient echo pulse; IR-FSPGR, fast spoiled gradient sequence with inversion preparation; MPRAGE, magnetization-prepared rapid acquisition with gradient echo sequence; MDEFT, modified driven equilibrium Fourier transform; SPGR, spoiled gradient recalled sequence; SPM, statistical parametric mapping; TFE, turbo field echo sequence; VBM, voxel-based morphometry.

Supplementary Table 2. MRI quality.

	Author	Year	MRI design described	Age reported	Sample gender reported	Sample handedness reported	Ethics approval reported	Image acquisition described	Image processing described	Statistical MRI-analysis described	Software package specified	Multiple comparison correction	Figures and tables
1	Agustus	2018	Y	Y	Y	Y	Y	Y	Y	Y	Y	Y	Y
2	Alluri	2012	Y	Y	Y	Y	Y	Y	Y	Y	Y	Y	Y
3	Alluri	2013	Y	Y	Y	Y	Y	Y	Y	Y	Y	Y	Y
4	Alonso	2016	Y	Y	Y	Y	Y	Y	Y	Y	Y	N	Y
5	Alttenmüller	2014	Y	Y	Y	Y	Y	Y	Y	Y	Y	Y	Y
6	Angulo-P	2014	Y	Y	Y	Y	Y	Y	Y	Y	Y	Y	Y
7	Armony	2015	Y	Y	Y	Y	Y	Y	Y	Y	Y	N	Y
8	Bangert	2006	Y	Y	Y	Y	Y	Y	Y	Y	Y	Y	Y
9	Barrett	2016	Y	Y	Y	N	Y	Y	Y	Y	Y	Y	Y
10	Barrett	2018	Y	N	N	N	Y	Y	Y	Y	Y	Y	Y
11	Barrett	2020	Y	Y	Y	N	Y	Y	Y	Y	Y	Y	Y
12	Bastepe-G	2020	Y	Y	Y	N	Y	Y	Y	Y	Y	Y	Y
13	Baumann	2007	Y	Y	Y	Y	Y	Y	Y	Y	Y	Y	Y
14	Bengtsson	2006	Y	Y	Y	Y	Y	Y	Y	Y	Y	Y	Y
15	Bengtsson	2007	Y	Y	Y	Y	Y	Y	Y	Y	Y	Y	Y
16	Bengtsson	2009	Y	Y	Y	Y	Y	Y	Y	Y	Y	Y	Y
17	Bianco	2016	Y	Y	Y	N	Y	Y	Y	Y	Y	N	Y
18	Bishop	2013	Y	Y	Y	N	Y	Y	Y	Y	Y	N	Y
19	Blood	1999	Y	N	N	N	N	Y	Y	Y	N	N	Y
20	Bodner	2001	Y	N	N	N	Y	Y	Y	Y	Y	N	Y
21	Bogert	2016	Y	Y	Y	Y	Y	Y	Y	Y	Y	Y	Y
22	Brattico	2016	Y	Y	Y	Y	Y	Y	Y	Y	Y	Y	Y
23	Bravo	2017a	Y	Y	Y	Y		Y	Y	Y	Y	Y	Y
24	Bravo	2017b	Y	Y	Y	Y	Y	Y	Y	Y	Y	Y	Y
25	Bravo	2020	Y	Y	Y	Y	Y	Y	Y	Y	Y	Y	Y
26	Brown	2004	Y	Y	Y	N	Y	Y	Y	Y	Y	Y	Y
27	Brown	2007	Y	Y	Y	N	Y	Y	N	N	N	N	Y
28	Chapin	2010	Y	Y	Y	N	Y	Y	Y	Y	Y	Y	Y
29	Chen	2008a	Y	N	N	N	Y	Y	Y	Y	Y	Y	Y
30	Chen	2008b	Y	Y	Y	N	Y	Y	Y	Y	Y	Y	Y
31	Chiang	2018	Y	Y	Y	Y	Y	Y	Y	Y	Y	Y	Y
32	Danielsen	2014	Y	Y	Y	N	Y	Y	Y	Y	Y	Y	Y
33	Demorest	2009	Y	Y	Y	Y	Y	Y	Y	Y	Y	Y	Y
34	Donnay	2014	Y	Y	Y	Y	Y	U	U	U	U	U	Y
35	Engel	2011	Y	Y	Y	Y	Y	Y	Y	Y	Y	Y	Y
36	Escoffier	2013	Y	Y	Y	Y	Y	Y	Y	Y	Y	Y	Y
37	Fedorenko	2012	Y	N	Y	N	Y	Y	Y	Y	Y	Y	Y
38	Flores-G	2007	Y	Y	Y	N	Y	Y	Y	Y	Y	Y	Y
39	Fujisawa	2011	Y	Y	Y	N	Y	Y	Y	Y	Y	N	Y
40	González-G	2016	Y	Y	Y	Y	Y	Y	Y	Y	Y	Y	Y
41	Grahn	2007	Y	Y	Y	N	Y	Y	Y	Y	Y	Y	Y
42	Grahn	2009a	Y	Y	Y	Y	Y	Y	Y	Y	Y	N	Y
43	Grahn	2009b	Y	Y	Y	Y	Y	Y	Y	Y	Y	Y	Y
44	Grahn	2013	Y	Y	Y	Y	Y	Y	Y	Y	Y	U	Y

45	Green	2008	Y	Y	N	N	Y	N	N	N	Y	N	N
46	Green	2012	Y	Y	Y	Y	Y	Y	Y	Y	Y	N	Y
47	Green	2018	Y	Y	Y	Y	Y	Y	Y	Y	Y	Y	Y
48	Halpern	1999	Y	Y	Y	N	Y	Y	N	N	N	N	Y
49	Halpern	2004	Y	Y	Y	Y	Y	Y	Y	Y	Y	N	Y
50	Herdener	2014	N	N	N	N	Y	N	N	Y	Y	Y	Y
51	Herholtz	2012	Y	Y	Y	Y	Y	Y	Y	Y	Y	Y	Y
52	Huang	2016	Y	N	Y	N	Y	Y	Y	Y	Y	Y	Y
53	Janata	2002a	Y	Y	Y	Y	Y	Y	Y	Y	Y	Y	Y
54	Janata	2002b	Y	Y	Y	Y	Y	Y	Y	Y	Y	Y	Y
55	Janata	2009	Y	Y	Y	N	Y	Y	Y	Y	Y	N	Y
56	Jeong	2011	Y	Y	Y	Y	Y	Y	Y	Y	Y	Y	Y
57	Jungblut	2012	Y	Y	N	Y	Y	Y	Y	Y	Y	Y	Y
58	Khalfa	2005	Y	Y	Y	Y	Y	Y	Y	Y	Y	Y	Y
59	Kleber	2007	Y	Y	Y	Y	Y	Y	Y	Y	Y	Y	Y
60	Kleider-O	2019	Y	Y	Y	Y	Y	Y	Y	Y	Y	Y	Y
61	Koelsch	2003	Y	Y	Y	N	Y	Y	Y	Y	Y	Y	Y
62	Koelsch	2014	Y	Y	Y	N	Y	Y	Y	Y	Y	Y	Y
63	Koelsch	2018	Y	Y	Y	Y	Y	Y	Y	Y	Y	Y	Y
64	Kornysheva	2010	Y	Y	Y	Y	Y	Y	Y	Y	Y	Y	Y
65	Langheim	2002	Y	Y	Y	Y	Y	Y	Y	Y	Y	Y	Y
66	Leaver	2009	Y	Y	Y	Y	Y	Y	Y	Y	Y	Y	Y
67	Lee	2011	Y	Y	Y	Y	Y	Y	Y	Y	Y	N	Y
68	Lehne	2013	Y	Y	Y	Y	Y	Y	Y	Y	Y	Y	Y
69	Levitin	2005	Y	Y	Y	Y	Y	Y	N	N	N	N	N
70	Levitin	2016	Y	Y	Y	Y	Y	Y	Y	Y	Y	N	Y
71	Li	2019	Y	Y	Y	Y	Y	Y	Y	Y	Y	Y	Y
72	Limb	2006	Y	Y	Y	Y	Y	Y	Y	Y	Y	Y	Y
73	Limb	2008	Y	Y	Y	Y	Y	Y	Y	Y	Y	Y	Y
74	Liu	2018	Y	Y	Y	N	Y	Y	Y	Y	Y	N	Y
75	Matthews	2020	Y	Y	Y	N	Y	Y	Y	Y	Y	Y	Y
76	Meister	2004	Y	Y	Y	Y	Y	Y	Y	Y	Y	N	Y
77	Merrill	2012	Y	Y	Y	Y	Y	Y	Y	Y	Y	Y	Y
78	Mizuno	2007	Y	Y	Y	Y	Y	Y	Y	Y	Y	Y	Y
79	Montag	2011	Y	Y	Y	N	Y	Y	Y	Y	Y	Y	Y
80	Morrison	2003	Y	Y	Y	N	Y	Y	Y	Y	Y	N	Y
81	Mueller	2015	Y	Y	Y	N	Y	Y	Y	Y	Y	Y	Y
82	Ohnishi	2001	Y	Y	Y	N	Y	Y	Y	Y	Y	N	Y
83	Park	2013	Y	Y	Y	Y	Y	Y	Y	Y	Y	Y	Y
84	Park	2014	Y	Y	Y	Y	Y	Y	Y	Y	Y	Y	Y
85	Parsons	2005	Y	Y	Y	Y	Y	Y	Y	Y	Y	N	Y
86	Pereira	2011	Y	Y	Y	Y	Y	Y	Y	Y	Y	Y	Y
87	Peretz	2009	Y	Y	Y	Y	Y	Y	Y	Y	Y	Y	Y
88	Petrini	2011	Y	Y	Y	N	Y	Y	Y	Y	Y	Y	Y
89	Pfordresher	2014	Y	Y	Y	Y	Y	Y	Y	Y	Y	Y	Y
90	Ragert	2014	Y	Y	Y	Y	Y	Y	Y	Y	Y	Y	Y
91	Reiterer	2008	Y	Y	Y	Y	Y	Y	Y	Y	Y	Y	Y
92	Rogalsky	2011	Y	Y	Y	Y	Y	Y	Y	Y	Y	Y	Y
93	Sammler	2010	Y	Y	Y	Y	Y	Y	Y	Y	Y	Y	Y
94	Schmithorst	2005	Y	Y	Y	Y	Y	Y	Y	Y	Y	Y	Y
95	Schön	2010	Y	Y	Y	Y	Y	U	Y	Y	Y	Y	Y
96	Schwenzer	2011	Y	U	Y	N	N	U	U	Y	Y	Y	Y
97	Shany	2019	Y	Y	Y	Y	Y	Y	Y	Y	Y	Y	Y

98	Sikka	2015	Y	Y	Y	Y	Y	Y	Y	Y	Y	Y	Y
99	Singer	2016	Y	Y	Y	Y	Y	Y	Y	Y	Y	Y	Y
100	Skouras	2014	Y	Y	Y	Y	Y	Y	Y	Y	Y	Y	Y
101	Spada	2014	Y	Y	Y	Y	Y	Y	Y	Y	Y	Y	Y
102	Tabei	2015	Y	Y	Y	Y	Y	Y	Y	Y	Y	Y	Y
103	Tachibana	2010	Y	Y	Y	Y	Y	Y	Y	Y	Y	Y	Y
104	Taruffi	2017	Y	Y	Y	Y	Y	Y	Y	Y	Y	Y	Y
105	Tervaniemi	2000	Y	Y	Y	Y	Y	Y	Y	Y	Y	Y	Y
106	Tervaniemi	2006	Y	Y	Y	Y	Y	Y	Y	Y	Y	N	Y
107	Thaut	2008	Y	Y	Y	Y	Y	Y	Y	Y	Y	Y	Y
108	Tillmann	2003	Y	Y	Y	Y	N	Y	Y	Y	Y	N	Y
109	Tillmann	2006	Y	Y	Y	Y	N	Y	Y	Y	Y	N	Y
110	Toiviainen	2014	Y	Y	Y	Y	Y	Y	Y	Y	Y	Y	Y
111	Trost	2012	Y	Y	Y	Y	Y	Y	Y	Y	Y	Y	Y
112	Trost	2014	Y	Y	Y	Y	Y	Y	Y	Y	Y	Y	N
113	Tsai	2010	Y	U	Y	Y	Y	Y	Y	Y	Y	N	Y
114	Tsai	2012	Y	Y	Y	N	Y	Y	Y	Y	Y	Y	Y
115	Tsai	2018	Y	Y	Y	N	Y	Y	Y	Y	Y	Y	Y
116	Tsai	2019	Y	Y	Y	N	Y	Y	Y	Y	Y	Y	Y
117	Uhlig	2013	Y	Y	Y	Y	Y	U	Y	Y	Y	Y	Y
118	Villarreal	2013	Y	Y	Y	Y	Y	Y	Y	Y	Y	Y	Y
119	Vuust	2011	Y	Y	Y	Y	Y	Y	Y	Y	Y	Y	Y
120	Wallmark	2018	Y	Y	Y	Y	Y	Y	Y	Y	Y	Y	Y
121	Watanabe	2008	Y	Y	Y	Y	Y	Y	Y	Y	Y	N	Y
122	Whitehead	2018	Y	Y	Y	Y	N	U	U	Y	Y	Y	Y
123	Wilson	2010	Y	Y	Y	Y	Y	Y	Y	Y	Y	Y	Y
124	Yoo	2001	Y	Y	Y	N	Y	U	U	Y	Y	N	Y
125	Zarate	2008	Y	N	Y	Y	Y	Y	U	Y	Y	Y	Y
126	Zarate	2010	Y	Y	Y	Y	Y	U	U	Y	Y	Y	Y
127	Zatorre	1994	Y	N	Y	Y	N	N	N	N	N	Y	Y
128	Zatorre	1996	Y	Y	Y	Y	N	N	N	U	Y	Y	Y
129	Zatorre	2010	Y	Y	Y	Y	Y	Y	Y	Y	Y	N	Y
130	Zvyagintsev	2013	Y	Y	Y	Y	Y	Y	Y	Y	Y	Y	Y

Y, yes; N, no; U, unclear.

Supplementary Table 3. Contrast analyses comparing music perception, production and imagery, at cluster level inference $p < 0.05$ (FWE).

Cluster number	Volume (mm^3)	MNI coordinates x	y	z	ALE	P	Z	Label (Side, region)
a. Perception + Production								
1	3448	66	-24	10	2E-02	-	-	R Superior Temporal Gyrus BA42
		50	-20	6	2E-02	-	-	R Superior Temporal Gyrus BA13
		64	-26	4	2E-02	-	-	R Superior Temporal Gyrus BA22
		62	-22	2	2E-02	-	-	R Superior Temporal Gyrus BA41
		50	-10	4	2E-02	-	-	R Insula BA13
		64	-28	14	2E-02	-	-	R Superior Temporal Gyrus BA42
		68	-16	8	2E-02	-	-	R Transverse Temporal Gyrus BA42
2	1816	-42	-28	6	3E-02	-	-	L Superior Temporal Gyrus BA13
3	152	0	-4	60	2E-02	-	-	L Medial Frontal Gyrus BA6
4	88	-4	6	54	2E-02	-	-	L Medial Frontal Gyrus BA6
5	24	50	10	24	1E-02	-	-	R Inferior Frontal Gyrus BA9
b. Perception - Production								
1	1096	-56	-16	-4	-	8E-03	2.4	L Superior Temporal Gyrus BA21
2	168	50	-18	-6	-	2E-02	2.1	R Superior Temporal Gyrus BA22
3	112	52	-10	-12	-	3E-02	1.9	R Superior Temporal Gyrus BA22
c. Production - Perception								
1	2816	-43.7	-23.7	45.4	-	1E-04	3.7	L Postcentral Gyrus BA2
		-42.7	-24	47.6	-	1E-04	3.7	L Postcentral Gyrus BA40
		-40	-21	44	-	7E-04	3.2	L Postcentral Gyrus BA2
		-44	-12	42	-	1E-03	3.0	L Precentral Gyrus BA4
		-34	-44	49	-	2E-03	2.9	L Inferior Parietal Lobule BA40
		-48	-10	42	-	3E-03	2.8	L Precentral Gyrus BA4
		-50	-6	40	-	3E-03	2.8	L Precentral Gyrus BA6
2	2128	-8	-7	51	-	0E+00	3.9	L Medial Frontal Gyrus BA6
		-12	-9	50	-	1E-04	3.7	L Medial Frontal Gyrus BA6
		-12	-7	54	-	2E-04	3.5	L Medial Frontal Gyrus BA6
		-4	-2	56	-	2E-03	2.9	L Medial Frontal Gyrus BA6
		4	0	54	-	3E-03	2.8	R Medial Frontal Gyrus BA6
		-2	6	44	-	1E-02	2.2	L Cingulate Gyrus BA24
3	1040	54.3	3.4	29.9	-	0E+00	3.9	R Precentral Gyrus BA6
4	608	-42	-34	4	-	8E-03	2.4	L Superior Temporal Gyrus BA41
		-42	-38	8	-	8E-03	2.4	L Superior Temporal Gyrus BA41
		-42	-28	6	-	1E-02	2.3	L Superior Temporal Gyrus BA13
		-41	-28	0	-	1E-02	2.3	L Insula BA13
5	584	-20	-60	-22	-	7E-04	3.2	L Cerebellum
6	536	56	8	-4	-	3E-03	2.8	R Superior Temporal Gyrus BA22
7	432	-50	4	0	-	7E-03	2.5	L Superior Temporal Gyrus BA22
8	424	2	22	38	-	3E-03	2.8	L Cingulate Gyrus BA32
9	400	-62	-42	20	-	2E-02	2.1	L Superior Temporal Gyrus BA22
10	352	68	-22	12	-	1E-02	2.3	R Superior Temporal Gyrus BA42
		66	-24	16	-	2E-02	2.1	R Superior Temporal Gyrus BA42
11	320	-30	0	2	-	2E-03	2.8	L Putamen
		-24	-4	2	-	7E-03	2.4	L Putamen
12	288	-60	-4	2	-	3E-03	2.8	L Superior Temporal Gyrus BA22
13	216	28	6	-10	-	6E-03	2.5	R Putamen
14	200	44	0	50	-	4E-03	2.6	R Middle Frontal Gyrus BA6
15	168	-4	14	62	-	6E-03	2.5	L Superior Frontal Gyrus BA6
16	120	-68	-18	6	-	2E-02	2.1	L Superior Temporal Gyrus BA22
17	112	-36	20	2	-	2E-02	2.0	L Insula BA13
d. Production + Imagery								
1	800	-2	-2	58	2E-02	-	-	L Medial Frontal Gyrus BA6
		-4	4	56	2E-02	-	-	L Medial Frontal Gyrus BA6
e. Production - Imagery								
1	1824	-36	-28	7	-	6E-04	3.2	L Superior Temporal Gyrus BA13
		-44	-30	8	-	9E-04	3.1	L Superior Temporal Gyrus BA41
		-40	-29.3	13.3	-	2E-03	2.9	L Superior Temporal Gyrus BA41
		-38	-36	12	-	2E-03	3.0	L Transverse Temporal Gyrus BA41
		-41	-24	12	-	4E-03	2.7	L Transverse Temporal Gyrus BA41
2	344	-42	-24	54	-	1E-02	2.2	L Postcentral Gyrus BA40
3	272	48	-24	4	-	8E-03	2.4	R Superior Temporal Gyrus BA22

		52	-26	4	-	2E-02	2.0	R Superior Temporal Gyrus BA41
4	240	-18	-60	-18	-	2E-02	2.0	L Cerebellum
		-20	-64	-20	-	3E-02	1.8	L Cerebellum
5	232	54	2	-2	-	8E-03	2.4	R Superior Temporal Gyrus BA22
f. Imagery - Production								
1	2552	-36.7	-58.7	59.3	-	4E-04	3.4	L Superior Parietal Lobule BA7
		-36	-64	56	-	1E-03	3.0	L Superior Parietal Lobule BA7
		-30	-54	38	-	4E-03	2.6	L Angular Gyrus BA39
2	760	0	4	62	-	5E-03	2.6	L Medial Frontal Gyrus BA6
3	696	56	6	48	-	9E-03	2.4	R Precentral Gyrus BA6
g. Imagery + Perception								
1	1848	0	6	58	2E-02	-	-	L Medial Frontal Gyrus BA6
		-2	0	62	2E-02	-	-	L Medial Frontal Gyrus BA6
2	624	54	0	50	2E-02	-	-	R Precentral Gyrus BA6
3	72	-20	-2	6	1E-02	-	-	L Lateral Globus Pallidus
h. Imagery - Perception								
1	3064	-32.6	-56.9	53.6	-	0E+00	3.9	L Inferior Parietal Lobule BA7
		-38	-60	50	-	2E-04	3.5	L Inferior Parietal Lobule BA7
		-32	-50	38	-	1E-03	3.0	L Inferior Parietal Lobule BA40
2	2672	-3.1	2.2	56.4	-	0E+00	3.9	L Medial Frontal Gyrus BA6
		2	5.3	56.7	-	1E-04	3.7	L Medial Frontal Gyrus BA6
3	1248	-16	-10	4	-	2E-03	2.8	L Thalamus
		-18	-18	8	-	3E-03	2.7	L Thalamus
		-18	-6	14	-	1E-02	2.2	L Thalamus
4	296	52	10	48	-	2E-03	3.0	R Middle Frontal Gyrus BA6
5	144	56	-2	54	-	2E-02	2.2	R Precentral Gyrus BA4
i. Perception - Imagery								
1	4936	46	-24	2	-	5E-04	3.3	R Superior Temporal Gyrus BA22
		52	-4	0	-	8E-03	2.4	R Superior Temporal Gyrus BA22
		40	-14	-2	-	2E-02	2.2	R Claustrum
		62	-26	-2	-	3E-02	1.9	R Superior Temporal Gyrus BA22
2	3272	-44	-28	12	-	3E-03	2.8	L Transverse Temporal Gyrus BA41
		-34	-30	8	-	4E-03	2.7	L Transverse Temporal Gyrus BA41
3	984	-44	-6	-6	-	8E-03	2.4	L Insula BA13
		-50	-10	-2	-	2E-02	2.0	L Superior Temporal Gyrus BA22
		-46	0	4	-	2E-02	2.0	L Insula BA13
4	544	-8	10	-4	-	7E-03	2.5	L Caudate Head

ALE, anatomic likelihood estimation; BA, Brodmann area; P, p-value; Z, peak z-value; R, right; L, left.

Supplementary Table 4. Meta-analytic connectivity modeling results from primary outcomes (n = 17 ROIs), at cluster level inference p < 0.05 (FWE).

Cluster number	Volume (mm³)	MNI coordinates x	y	z	ALE	P	Z	Label (Side, region)
**1. Music perception**								
a. STG BA22 R: 824 foci, 43 experiments, 610 subjects (x=52, y=-20, z=4)								
1	13592	-54	-18	2	6E-02	4E-20	9.1	L Superior Temporal Gyrus BA22
		-58	-28	6	5E-02	9E-15	7.7	L Superior Temporal Gyrus BA22
		-42	-30	8	5E-02	5E-13	7.1	L Superior Temporal Gyrus BA41
		-48	-36	14	4E-02	7E-11	6.4	L Superior Temporal Gyrus BA41
2	10480	52	-20	4	2E-01	0E+00	19.9	R Superior Temporal Gyrus BA13
		60	-6	2	3E-02	7E-07	4.8	R Superior Temporal Gyrus BA22
3	4312	34	20	2	4E-02	1E-10	6.3	R Claustrum
		50	10	2	3E-02	8E-07	4.8	R Insula BA13
		46	8	2	3E-02	2E-06	4.6	R Precentral Gyrus BA44
		54	18	-4	2E-02	2E-05	4.1	R Inferior Frontal Gyrus
4	4088	-2	2	62	4E-02	1E-10	6.3	L Medial Frontal Gyrus BA6
		4	10	54	3E-02	3E-08	5.4	R Superior Frontal Gyrus BA6
		4	16	40	2E-02	1E-05	4.2	R Cingulate Gyrus BA32
		-2	12	44	2E-02	2E-05	4.1	L Medial Frontal Gyrus BA32
5	2368	50	-6	38	3E-02	4E-08	5.4	R Precentral Gyrus BA6
		42	6	40	2E-02	1E-04	3.6	R Middle Frontal Gyrus BA6
6	2056	-54	12	-4	3E-02	6E-09	5.7	L Superior Temporal Gyrus BA22
		-52	2	-8	2E-02	2E-04	3.6	L Superior Temporal Gyrus BA22
7	1512	-38	16	4	3E-02	1E-07	5.2	L Insula BA13
8	1048	-12	-64	-16	3E-02	8E-07	4.8	L Cerebellum
		-26	-66	-18	2E-02	3E-05	4.0	L Cerebellum
b. STG BA22 L: 1143 foci, 60 experiments, 850 subjects (x=-54, y=-16, z=2)								
1	19976	-54	-16	2	3E-01	0E+00	24.2	L Superior Temporal Gyrus BA22
		-62	-30	8	6E-02	4E-15	7.8	L Superior Temporal Gyrus BA42
		-60	-34	12	6E-02	7E-15	7.7	L Superior Temporal Gyrus BA22
		-44	-34	14	4E-02	2E-08	5.5	L Superior Temporal Gyrus BA41
		-52	-40	20	3E-02	2E-06	4.6	L Superior Temporal Gyrus BA13
		-50	2	-8	3E-02	4E-06	4.5	L Superior Temporal Gyrus BA22
		-50	10	-6	3E-02	8E-06	4.3	L Insula BA13
		-44	2	0	2E-02	2E-05	4.1	L Insula BA13
		-54	6	8	2E-02	5E-05	3.9	L Precentral Gyrus BA44
2	19720	58	-14	0	1E-01	3E-36	12.5	R Superior Temporal Gyrus BA22
		64	-32	8	5E-02	5E-14	7.4	R Superior Temporal Gyrus BA42
		54	12	-12	3E-02	2E-06	4.6	R Superior Temporal Gyrus BA22
3	1824	36	22	4	3E-02	4E-06	4.5	R Insula BA13
		36	22	-8	2E-02	2E-05	4.1	R Insula
c. MedFG BA6 L: 1773 foci, 58 experiments, 838 subjects (x=-2, y=-2, z=66)								
1	28752	-2	-2	64	3E-01	0E+00	22.8	L Medial Frontal Gyrus BA6
		52	2	50	5E-02	8E-12	6.7	R Precentral Gyrus BA6
		36	24	2	5E-02	6E-10	6.1	R Insula BA13
		-6	12	42	4E-02	7E-09	5.7	L Cingulate Gyrus BA32
		56	8	38	4E-02	7E-09	5.7	R Middle Frontal Gyrus BA6
		8	12	38	4E-02	1E-08	5.6	R Cingulate Gyrus BA32
		42	-4	54	4E-02	1E-07	5.1	R Precentral Gyrus BA6
		30	-6	60	4E-02	2E-07	5.0	R Middle Frontal Gyrus BA6
		46	8	4	4E-02	3E-07	5.0	R Insula BA13
		54	14	14	3E-02	8E-06	4.3	R Inferior Frontal Gyrus BA44
		62	10	16	3E-02	8E-06	4.3	R Inferior Frontal Gyrus BA44
		52	-8	38	3E-02	8E-05	3.8	R Precentral Gyrus BA4
		18	-4	66	3E-02	2E-04	3.5	R Superior Frontal Gyrus BA6
		6	30	36	2E-02	5E-04	3.3	R Cingulate Gyrus BA32
2	19104	-50	-8	50	5E-02	1E-11	6.7	L Precentral Gyrus BA4
		-30	22	4	5E-02	7E-10	6.1	L Insula BA13
		-54	2	38	5E-02	2E-09	5.9	L Precentral Gyrus BA6
		-30	-8	56	5E-02	3E-09	5.8	L Precentral Gyrus BA6

		-54	12	0	4E-02	5E-09	5.7	L Superior Temporal Gyrus BA22
		-54	8	20	4E-02	2E-07	5.0	L Inferior Frontal Gyrus BA44
		-38	-26	54	3E-02	3E-05	4.0	L Postcentral Gyrus BA3
		-48	12	28	3E-02	5E-05	3.9	L Inferior Frontal Gyrus BA9
		-60	-4	20	2E-02	3E-04	3.4	L Precentral Gyrus BA4
3	8864	-22	2	4	7E-02	1E-15	7.9	L Lentiform Nucleus
		-12	-18	4	5E-02	7E-12	6.8	L Thalamus
4	8048	16	-16	6	6E-02	8E-15	7.7	R Thalamus
		22	6	4	6E-02	1E-13	7.3	R Lentiform Nucleus
5	5008	8	-66	-18	4E-02	1E-08	5.6	R Declive
		18	-60	-20	4E-02	2E-08	5.5	R Culmen
		32	-62	-26	4E-02	1E-07	5.2	R Culmen
		38	-68	-24	3E-02	2E-06	4.6	R Declive
		24	-74	-20	3E-02	1E-04	3.7	R Declive
6	4584	66	-32	14	4E-02	8E-08	5.2	R Superior Temporal Gyrus BA22
		58	-14	4	4E-02	3E-07	5.0	R Superior Temporal Gyrus BA22
		56	-22	4	3E-02	4E-06	4.4	R Superior Temporal Gyrus BA41
		66	-22	4	3E-02	1E-05	4.2	R Superior Temporal Gyrus BA41
		60	-6	4	3E-02	5E-05	3.9	R Superior Temporal Gyrus BA22
7	4056	-28	-60	-26	5E-02	4E-12	6.8	L Culmen
		-12	-52	-22	3E-02	5E-06	4.4	L Dentate
		-12	-64	-18	3E-02	7E-06	4.3	L Declive
		-16	-64	-18	3E-02	9E-06	4.3	L Declive
8	2360	-30	-54	54	4E-02	3E-07	5.0	L Superior Parietal Lobule BA7
		-22	-60	58	3E-02	3E-06	4.5	L Precuneus BA7
9	2320	-60	-32	8	3E-02	2E-06	4.6	L Superior Temporal Gyrus BA42
		-64	-34	10	3E-02	2E-06	4.6	L Superior Temporal Gyrus BA22
		-52	-42	20	3E-02	1E-05	4.2	L Superior Temporal Gyrus BA13
		-58	-22	16	3E-02	2E-04	3.6	L Postcentral Gyrus BA40
d. PUT R: 1821 foci, 70 experiments, 1052 subjects (x=22, y=8, z=6)								
1	34416	-22	4	6	1E-01	3E-28	11.0	L Lentiform Nucleus
		-30	20	2	8E-02	3E-18	8.6	L Claustrum
		-12	-14	6	7E-02	7E-16	8.0	L Thalamus
		-52	8	18	5E-02	2E-10	6.3	L Inferior Frontal Gyrus BA44
		-30	-8	56	5E-02	3E-09	5.8	L Precentral Gyrus BA6
		-46	4	4	5E-02	7E-09	5.7	L Precentral Gyrus BA44
		-44	6	26	4E-02	4E-08	5.4	L Inferior Frontal Gyrus BA9
		-46	-6	54	4E-02	4E-08	5.4	L Precentral Gyrus BA4
		-54	6	34	4E-02	1E-07	5.2	L Precentral Gyrus BA6
		-48	-2	38	3E-02	5E-06	4.4	L Precentral Gyrus BA6
		-48	-10	44	3E-02	1E-05	4.2	L Precentral Gyrus BA4
		-30	-14	-2	2E-02	7E-04	3.2	L Lentiform Nucleus
2	29528	22	8	6	3E-01	0E+00	24.2	R Lentiform Nucleus
		36	20	2	6E-02	9E-12	6.7	R Insula BA13
		14	-12	6	5E-02	3E-10	6.2	R Thalamus
		54	2	46	5E-02	8E-10	6.0	R Precentral Gyrus BA6
		58	8	24	4E-02	1E-08	5.6	R Inferior Frontal Gyrus BA9
		54	6	12	4E-02	2E-07	5.1	R Inferior Frontal Gyrus BA44
		14	-16	14	4E-02	8E-07	4.8	R Thalamus
		48	12	2	3E-02	8E-06	4.3	R Precentral Gyrus BA44
		46	8	-4	3E-02	3E-05	4.0	R Insula
3	11672	-2	0	62	7E-02	8E-15	7.7	L Medial Frontal Gyrus BA6
		-4	2	58	6E-02	4E-14	7.5	L Medial Frontal Gyrus BA6
		8	2	60	6E-02	1E-13	7.3	R Medial Frontal Gyrus BA6
		-2	6	50	6E-02	2E-12	7.0	L Medial Frontal Gyrus BA6
		8	14	40	4E-02	3E-07	5.0	R Cingulate Gyrus BA32
4	3472	42	-44	46	4E-02	3E-08	5.4	R Inferior Parietal Lobule BA40
		44	-38	46	4E-02	3E-07	5.0	R Inferior Parietal Lobule BA40
		58	-30	42	3E-02	4E-05	3.9	R Inferior Parietal Lobule BA40
		40	-40	58	3E-02	1E-04	3.6	R Inferior Parietal Lobule BA40
5	1784	36	42	24	4E-02	2E-07	5.1	R Middle Frontal Gyrus BA9
e. PUT L: 1493 foci, 69 experiments, 1139 subjects (x=-22, y=4, z=6)								
1	31472	-22	4	6	3E-01	0E+00	25.1	L Lentiform Nucleus
		-34	24	0	6E-02	2E-13	7.3	L Insula BA13
		-12	-16	6	6E-02	2E-13	7.2	L Thalamus
		-52	-4	46	4E-02	2E-08	5.5	L Precentral Gyrus BA4

		-50	6	32	4E-02	3E-08	5.4	L Precentral Gyrus BA6
		-54	6	6	4E-02	6E-08	5.3	L Superior Temporal Gyrus BA22
		-54	12	2	4E-02	1E-07	5.1	L Precentral Gyrus BA44
		-52	10	18	3E-02	9E-07	4.8	L Inferior Frontal Gyrus BA44
		-40	34	12	2E-02	2E-04	3.5	L Middle Frontal Gyrus BA46
		-30	-12	-2	2E-02	2E-04	3.5	L Lentiform Nucleus
		-10	-18	-10	2E-02	5E-04	3.3	L Brainstem
2	17664	22	6	6	1E-01	8E-32	11.7	R Lentiform Nucleus
		34	18	4	6E-02	2E-14	7.6	R Claustrum
		14	-14	4	5E-02	5E-10	6.1	R Thalamus
		34	26	-8	3E-02	2E-06	4.6	R Insula BA13
		50	14	0	3E-02	3E-05	4.0	R Insula BA13
		54	8	6	3E-02	1E-04	3.7	R Precentral Gyrus BA44
3	11768	-4	0	62	7E-02	2E-18	8.7	L Medial Frontal Gyrus BA6
		8	16	42	5E-02	5E-10	6.1	R Cingulate Gyrus BA32
		8	4	60	4E-02	1E-07	5.1	R Medial Frontal Gyrus BA6
		-8	12	38	3E-02	1E-05	4.2	L Cingulate Gyrus BA32
		4	16	52	3E-02	2E-05	4.1	R Superior Frontal Gyrus BA6
		8	28	38	2E-02	4E-04	3.4	R Cingulate Gyrus BA32
4	3632	54	-32	4	4E-02	4E-07	4.9	R Superior Temporal Gyrus BA22
		54	-26	0	3E-02	8E-07	4.8	R Superior Temporal Gyrus BA22
		66	-22	6	3E-02	5E-06	4.4	R Superior Temporal Gyrus BA42
		68	-32	14	2E-02	4E-04	3.4	R Superior Temporal Gyrus BA22
5	3624	54	2	46	5E-02	1E-10	6.3	R Precentral Gyrus BA6
		48	10	28	3E-02	3E-06	4.5	R Inferior Frontal Gyrus BA9
		56	10	34	3E-02	4E-05	3.9	R Middle Frontal Gyrus BA9
6	2920	-28	-60	-26	6E-02	8E-13	7.1	L Culmen
7	2768	-52	-42	18	3E-02	6E-06	4.4	L Superior Temporal Gyrus BA13
		-62	-30	8	3E-02	3E-05	4.0	L Superior Temporal Gyrus BA42
		-60	-16	6	3E-02	5E-05	3.9	L Superior Temporal Gyrus BA41
		-60	-36	14	3E-02	5E-05	3.9	L Superior Temporal Gyrus BA22
		-54	-40	12	3E-02	6E-05	3.8	L Superior Temporal Gyrus BA22
		-52	-16	2	3E-02	1E-04	3.7	L Superior Temporal Gyrus BA22
f. CRBL L: 1201 foci, 47 experiments, 688 subjects (x=-28, y=-64, z=-26)								
1	11472	36	18	6	4E-02	3E-09	5.8	R Insula BA13
		42	24	-2	4E-02	4E-09	5.8	R Insula BA13
		48	18	-2	4E-02	7E-08	5.3	R Insula BA13
		56	4	34	3E-02	1E-07	5.2	R Precentral Gyrus BA6
		54	0	46	3E-02	2E-07	5.0	R Precentral Gyrus BA4
		32	24	-8	3E-02	5E-07	4.9	R Claustrum
		52	12	14	3E-02	2E-06	4.6	R Insula BA13
		50	10	22	3E-02	3E-05	4.0	R Inferior Frontal Gyrus BA9
		38	10	-8	2E-02	8E-05	3.8	R Claustrum
		52	-6	36	2E-02	4E-04	3.4	R Precentral Gyrus BA6
2	10608	30	-62	-28	8E-02	5E-22	9.6	R Culmen
		10	-74	-20	4E-02	5E-09	5.7	R Declive
		-6	-74	-28	3E-02	8E-07	4.8	L Uvula
		2	-58	-22	3E-02	2E-05	4.1	R.Culmen
3	9624	0	14	44	4E-02	1E-10	6.3	L Medial Frontal Gyrus BA32
		8	16	44	4E-02	6E-10	6.1	R Medial Frontal Gyrus BA32
		-4	2	60	4E-02	2E-09	5.9	L Medial Frontal Gyrus BA6
		6	10	56	4E-02	3E-09	5.8	R Medial Frontal Gyrus BA6
4	9448	-34	22	2	5E-02	3E-13	7.2	L Insula BA13
		-22	4	6	5E-02	6E-13	7.1	L Lentiform Nucleus
		-12	-14	4	5E-02	6E-11	6.4	L Thalamus
5	7488	-58	-16	6	4E-02	3E-08	5.4	L Superior Temporal Gyrus BA41
		-42	-34	12	3E-02	1E-07	5.2	L Transverse Temporal Gyrus BA41
		-54	-40	12	3E-02	2E-07	5.1	L Superior Temporal Gyrus BA22
		-62	-34	12	3E-02	3E-07	5.0	L Superior Temporal Gyrus BA22
		-60	-36	24	3E-02	2E-06	4.6	L Insula BA13
6	6056	-28	-62	-26	2E-01	0E+00	19.7	L Culmen
7	3568	58	-14	2	3E-02	8E-07	4.8	R Superior Temporal Gyrus BA22
		58	-32	4	3E-02	6E-06	4.4	R Middle Temporal Gyrus BA22
		56	-28	0	3E-02	6E-06	4.4	R Superior Temporal Gyrus
		54	-24	6	3E-02	2E-05	4.1	R Superior Temporal Gyrus BA41
		66	-22	6	2E-02	6E-05	3.8	R Superior Temporal Gyrus BA42

		68	-32	12	2E-02	1E-04	3.7	R Superior Temporal Gyrus BA22
8	2256	-54	10	2	4E-02	8E-09	5.6	L Precentral Gyrus BA44
9	2152	22	8	6	4E-02	3E-10	6.2	R Lentiform Nucleus
10	2024	12	-14	6	4E-02	2E-08	5.5	R Thalamus
		6	-24	-2	2E-02	8E-05	3.8	R Thalamus
g. INS L: 1787 foci, 87 experiments, 1332 subjects (x=-32, y=18, z=10)								
1	45704	-32	18	8	4E-01	0E+00	29.1	L Insula BA13
		36	22	6	1E-01	1E-42	13.6	R Insula BA13
		-10	-16	8	9E-02	1E-24	10.2	L Thalamus
		-52	8	22	6E-02	6E-13	7.1	L Inferior Frontal Gyrus BA9
		10	-16	6	5E-02	2E-11	6.6	R Thalamus
		18	10	4	5E-02	1E-09	6.0	R Caudate
		-18	6	6	4E-02	1E-08	5.5	L Lentiform Nucleus
		-44	4	-2	4E-02	1E-07	5.2	L Insula BA13
		-46	0	4	4E-02	2E-07	5.1	L Insula BA13
		-38	-4	14	4E-02	3E-07	5.0	L Insula BA13
		8	-22	-10	3E-02	2E-04	3.5	R Brainstem
2	12360	-2	10	50	7E-02	2E-15	7.8	L Medial Frontal Gyrus BA6
		10	16	42	4E-02	2E-08	5.5	R Medial Frontal Gyrus BA32
		-6	16	36	4E-02	8E-07	4.8	L Cingulate Gyrus BA32
		0	30	32	3E-02	6E-05	3.9	L Cingulate Gyrus BA32
		-2	26	44	3E-02	3E-04	3.4	L Medial Frontal Gyrus BA8
3	6064	50	10	28	9E-02	1E-21	9.5	R Inferior Frontal Gyrus BA9
		56	16	16	3E-02	1E-04	3.7	R Inferior Frontal Gyrus BA44
4	3864	-34	-48	44	4E-02	6E-07	4.8	L Inferior Parietal Lobule BA40
		-44	-38	50	4E-02	1E-06	4.7	L Inferior Parietal Lobule BA40
		-30	-56	52	3E-02	3E-05	4.0	L Superior Parietal Lobule BA7
		-24	-66	48	3E-02	4E-05	4.0	L Superior Parietal Lobule BA7
5	3360	-30	-8	56	5E-02	1E-09	6.0	L Precentral Gyrus BA6
		-42	-4	48	3E-02	2E-04	3.5	L Precentral Gyrus BA6
6	3200	44	42	18	4E-02	2E-06	4.7	R Middle Frontal Gyrus BA10
		42	38	28	3E-02	4E-06	4.5	R Middle Frontal Gyrus BA9
		40	50	12	3E-02	1E-04	3.7	R Middle Frontal Gyrus BA10
7	2744	-34	42	26	4E-02	2E-06	4.6	L Superior Frontal Gyrus BA9
		-42	38	12	3E-02	4E-06	4.5	L Middle Frontal Gyrus BA46
		-34	50	12	3E-02	5E-05	3.9	L Middle Frontal Gyrus BA10
		-42	32	26	3E-02	1E-04	3.6	L Middle Frontal Gyrus BA9
8	2416	34	2	62	4E-02	1E-08	5.6	R Middle Frontal Gyrus BA6
		34	2	50	3E-02	9E-06	4.3	R Middle Frontal Gyrus BA6
h. PreCG R: 1613 foci, 63 experiments, 931 subjects (x=54, y=0, z=46)								
1	20416	54	0	46	3E-01	0E+00	23.8	R Precentral Gyrus BA4
		34	24	4	5E-02	2E-10	6.3	R Insula BA13
		38	22	-2	5E-02	1E-09	6.0	R Insula BA13
		44	14	6	4E-02	2E-08	5.5	R Insula BA13
		50	14	2	4E-02	5E-08	5.3	R Precentral Gyrus BA44
		36	-4	58	4E-02	6E-08	5.3	R Precentral Gyrus BA6
		52	10	22	4E-02	1E-07	5.1	R Inferior Frontal Gyrus BA9
		52	6	28	4E-02	3E-07	5.0	R Precentral Gyrus BA6
		60	6	12	3E-02	3E-06	4.5	R Precentral Gyrus BA6
		58	8	-6	3E-02	7E-05	3.8	R Superior Temporal Gyrus BA22
2	17984	-50	-6	48	9E-02	6E-24	10.0	L Precentral Gyrus BA4
		-34	20	4	5E-02	2E-11	6.6	L Insula BA13
		-54	8	24	5E-02	2E-09	5.9	L Inferior Frontal Gyrus BA9
		-58	0	16	4E-02	9E-09	5.6	L Precentral Gyrus BA4
		-56	6	6	4E-02	2E-08	5.5	L Superior Temporal Gyrus BA22
		-50	12	0	4E-02	7E-08	5.3	L Insula BA13
3	14664	-2	0	60	9E-02	3E-23	9.9	L Medial Frontal Gyrus BA6
		-2	20	38	4E-02	2E-08	5.5	L Cingulate Gyrus BA32
		8	14	38	4E-02	5E-07	4.9	R Cingulate Gyrus BA32
4	6080	-54	-18	0	4E-02	1E-08	5.6	L Superior Temporal Gyrus BA22
		-54	-40	22	4E-02	6E-08	5.3	L Superior Temporal Gyrus BA13
		-62	-30	8	4E-02	7E-08	5.3	L Superior Temporal Gyrus BA42
5	5912	56	-32	4	4E-02	3E-08	5.4	R Superior Temporal Gyrus BA22
		64	-34	26	4E-02	5E-07	4.9	R Inferior Parietal Lobule BA40
		60	-18	4	3E-02	3E-06	4.5	R Superior Temporal Gyrus BA41
6	5056	12	-14	6	4E-02	3E-09	5.8	R Thalamus

		24	4	4	4E-02	2E-08	5.5	R Putamen
		24	0	6	4E-02	1E-07	5.1	R Putamen
7	1984	-22	4	6	5E-02	2E-09	5.9	L Putamen
		-28	-6	-4	3E-02	5E-05	3.9	L Putamen
		-22	-2	16	2E-02	3E-04	3.5	L Putamen
**2. Music production**								
a. STG BA42 R: 753 foci, 32 experiments, 455 subjects (x=66, y=-24, z=10)								
1	7648	64	-24	10	1E-01	0E+00	17.2	R Superior Temporal Gyrus BA41
		54	-16	4	2E-02	9E-05	3.7	R Superior Temporal Gyrus BA22
		62	-40	2	2E-02	2E-04	3.6	R Middle Temporal Gyrus BA22
2	7224	-60	-24	10	5E-02	3E-15	7.8	L Superior Temporal Gyrus BA41
		-42	-34	14	2E-02	1E-04	3.7	L Transverse Temporal Gyrus BA41
		-42	-26	12	1E-02	9E-04	3.1	L Transverse Temporal Gyrus BA41
3	3192	-4	6	52	3E-02	1E-08	5.6	L Medial Frontal Gyrus BA6
		-4	-2	62	2E-02	1E-06	4.7	L Medial Frontal Gyrus BA6
		2	0	70	2E-02	5E-05	3.9	L Medial Frontal Gyrus BA6
		2	16	48	2E-02	7E-05	3.8	L Medial Frontal Gyrus BA32
4	2736	-34	24	0	3E-02	7E-07	4.8	L Insula BA13
		-52	8	-2	3E-02	1E-06	4.7	L Superior Temporal Gyrus BA22
		-48	14	0	2E-02	6E-06	4.4	L Insula BA13
		-52	0	4	2E-02	6E-04	3.2	L Precentral Gyrus BA44
5	2624	52	4	-8	3E-02	2E-08	5.5	R Superior Temporal Gyrus BA22
		48	20	-6	2E-02	4E-05	4.0	R Insula
6	1672	-12	-22	-6	2E-02	6E-06	4.4	L Brainstem
		-12	-18	0	2E-02	7E-06	4.3	L Thalamus
		-10	-12	4	2E-02	6E-05	3.8	L Thalamus
7	1608	54	0	44	3E-02	5E-07	4.9	R Precentral Gyrus BA6
		54	8	30	2E-02	7E-05	3.8	R Inferior Frontal Gyrus BA9
b. PreCG L: 1372 foci, 79 experiments, 1034 subjects (x=-48, y=0, z=42)								
1	25824	-48	0	42	3E-01	0E+00	26.8	L Precentral Gyrus BA6
		-32	18	2	6E-02	1E-13	7.4	L Claustrum
		-56	8	18	5E-02	2E-12	6.9	L Inferior Frontal Gyrus BA44
		-46	16	22	5E-02	3E-12	6.9	L Inferior Frontal Gyrus BA9
		-22	-8	58	4E-02	5E-09	5.7	L Middle Frontal Gyrus BA6
		-32	-6	52	2E-02	7E-05	3.8	L Precentral Gyrus BA6
		-42	28	-2	2E-02	8E-05	3.8	L Inferior Frontal Gyrus BA47
		-56	-6	-6	2E-02	1E-04	3.7	L Superior Temporal Gyrus BA22
2	13904	-2	10	50	9E-02	2E-24	10.1	L Medial Frontal Gyrus BA6
		0	6	62	7E-02	9E-18	8.5	L Medial Frontal Gyrus BA6
3	8792	52	2	36	5E-02	2E-10	6.3	R Precentral Gyrus BA6
		50	10	34	4E-02	9E-10	6.0	R Precentral Gyrus BA9
		38	0	54	4E-02	1E-07	5.1	R Middle Frontal Gyrus BA6
		42	16	24	3E-02	8E-06	4.3	R Middle Frontal Gyrus BA9
4	7520	-36	-52	44	5E-02	2E-11	6.6	L Inferior Parietal Lobule BA40
		-42	-38	42	3E-02	3E-06	4.5	L Inferior Parietal Lobule BA40
		-26	-64	44	3E-02	2E-05	4.1	L Precuneus BA7
5	3016	-60	-36	4	5E-02	1E-10	6.3	L Middle Temporal Gyrus BA22
		-60	-26	4	3E-02	1E-06	4.7	L Superior Temporal Gyrus BA22
		-54	-38	14	2E-02	1E-04	3.6	L Superior Temporal Gyrus BA41
6	2760	-40	-76	-10	4E-02	1E-08	5.6	L Fusiform Gyrus BA19
		-38	-70	-12	3E-02	6E-07	4.9	L.Declive
		-42	-60	-8	3E-02	7E-06	4.4	L Fusiform Gyrus BA37
7	2608	28	-60	54	3E-02	3E-06	4.6	R Superior Parietal Lobule BA7
		36	-54	50	3E-02	5E-06	4.4	R Inferior Parietal Lobule BA7
		40	-44	44	3E-02	7E-06	4.3	R Inferior Parietal Lobule BA40
8	2208	36	-62	-28	3E-02	2E-06	4.6	R Culmen
		30	-72	-18	3E-02	1E-05	4.3	R Declive
9	1880	36	20	0	4E-02	6E-08	5.3	R Claustrum
		48	16	2	2E-02	9E-05	3.8	R Precentral Gyrus BA44
c. MedFG BA6 L: 403 foci, 21 experiments, 261 subjects (x=-10, y=-10, z=52)								
1	11352	-36	-24	58	4E-02	2E-14	8E+00	L Precentral Gyrus BA4
		-36	-34	46	3E-02	2E-08	6E+00	L Inferior Parietal Lobule BA40
		-48	-18	52	2E-02	1E-07	5E+00	L Postcentral Gyrus BA2
2	6112	-8	-8	52	1E-01	0E+00	1E+01	L Medial Frontal Gyrus BA6
		8	-12	50	2E-02	6E-06	4E+00	R Paracentral Lobule BA31

3	2280	22	-52	-24	2E-02	4E-08	5E+00	R Culmen
		14	-56	-14	1E-02	5E-04	3E+00	R Culmen
4	1952	-56	-24	22	2E-02	1E-06	5E+00	L Postcentral Gyrus BA40
		-46	-28	20	2E-02	3E-06	5E+00	L Insula BA13
		-52	-28	22	2E-02	4E-06	4E+00	L Insula BA13
5	1816	-38	-2	4	2E-02	1E-06	5E+00	L Claustrum
		-44	-4	4	2E-02	2E-05	4E+00	L Insula BA13
		-30	-14	6	1E-02	1E-04	4E+00	L Lentiform Nucleus
		-30	-8	6	1E-02	2E-04	4E+00	L Lentiform Nucleus
6	1224	-16	-22	6	2E-02	2E-07	5E+00	L Thalamus
d. STG BA41 L: 529 foci, 38 experiments, 522 subjects (x=-42, y=-28, z=6)								
1	13576	50	-26	6	5E-02	2E-17	8.4	R Superior Temporal Gyrus BA41
		50	-18	4	5E-02	4E-17	8.3	R Superior Temporal Gyrus BA13
		66	-30	4	4E-02	4E-12	6.9	R Middle Temporal Gyrus BA22
2	11104	-42	-28	6	2E-01	0E+00	17.6	L Superior Temporal Gyrus BA13
		-50	-14	0	3E-02	1E-08	5.6	L Superior Temporal Gyrus BA22
		-62	-30	12	3E-02	8E-08	5.2	L Superior Temporal Gyrus BA42
		-60	-24	6	3E-02	1E-07	5.2	L Superior Temporal Gyrus BA41
3	1248	-28	-10	-4	3E-02	2E-08	5.5	L Putamen
e. PreCG R: 1105 foci, 51 experiments, 846 subjects (x=54, y=4, z=32)								
1	11192	-2	10	48	5E-02	1E-13	7.3	L Cingulate Gyrus BA24
		6	16	42	5E-02	7E-13	7.1	R Cingulate Gyrus BA32
		4	8	50	5E-02	6E-11	6.4	R Medial Frontal Gyrus BA6
		-2	-2	62	4E-02	1E-08	5.5	L Medial Frontal Gyrus BA6
2	9016	54	4	32	2E-01	0E+00	20.8	R Precentral Gyrus BA6
		56	10	12	3E-02	2E-06	4.6	R Inferior Frontal Gyrus BA44
3	8128	-50	6	28	5E-02	5E-13	7.1	L Precentral Gyrus BA6
		-50	2	42	5E-02	5E-11	6.5	L Precentral Gyrus BA6
4	2792	-28	-62	-26	3E-02	2E-06	4.7	L Culmen
		-42	-58	-18	3E-02	3E-06	4.5	L Fusiform Gyrus BA37
		-14	-62	-18	3E-02	7E-06	4.4	L Declive
5	2600	-28	-52	54	3E-02	1E-07	5.1	L Superior Parietal Lobule BA7
		-24	-60	54	3E-02	1E-06	4.8	L Superior Parietal Lobule BA7
6	2480	-58	-34	28	3E-02	6E-06	4.4	L Inferior Parietal Lobule BA40
		-50	-28	38	3E-02	2E-05	4.1	L Postcentral Gyrus BA2
		-38	-28	52	2E-02	4E-05	4.0	L Postcentral Gyrus BA3
		-46	-30	24	2E-02	9E-05	3.7	L Insula BA13
7	1816	36	22	4	4E-02	3E-09	5.8	R Insula BA13
8	1816	30	-56	50	3E-02	1E-07	5.1	R Superior Parietal Lobule BA7
		24	-60	60	2E-02	3E-04	3.4	R Superior Parietal Lobule BA7
9	1656	60	-32	24	3E-02	1E-06	4.7	R Inferior Parietal Lobule BA40
		50	-32	30	2E-02	4E-05	3.9	R Inferior Parietal Lobule BA40
		62	-22	28	2E-02	5E-05	3.9	R Inferior Parietal Lobule BA40
10	1408	10	-18	8	4E-02	9E-10	6.0	R Thalamus
**3. Music imagery**								
a. MedFG L: 3287 foci, 161 experiments, 2114 subjects (x=0, y=6, z=58)								
1	59384	-34	24	0	1E-01	1E-26	10.6	L Insula BA13
		-48	4	34	1E-01	3E-24	10.1	L Precentral Gyrus BA6
		-36	-4	54	9E-02	1E-19	9.0	L Precentral Gyrus BA6
		-40	-4	54	9E-02	2E-19	9.0	L Precentral Gyrus BA6
		-48	-4	48	9E-02	2E-19	8.9	L Precentral Gyrus BA4
		-10	-18	6	9E-02	4E-17	8.3	L Thalamus
		-50	12	0	9E-02	5E-17	8.3	L Insula BA13
		10	-16	6	7E-02	2E-11	6.6	R Thalamus
		20	2	2	6E-02	2E-10	6.3	R Lentiform Nucleus
		-24	2	-2	6E-02	2E-10	6.2	L Lentiform Nucleus
		-22	4	2	6E-02	3E-10	6.2	L Lentiform Nucleus
		-24	-4	6	6E-02	2E-09	5.9	L Lentiform Nucleus
		-12	-2	6	6E-02	4E-09	5.7	L Thalamus
		-48	28	24	4E-02	9E-06	4.3	L Middle Frontal Gyrus BA46
		-56	-20	22	4E-02	2E-05	4.1	L Postcentral Gyrus BA40
		-58	-16	26	4E-02	7E-05	3.8	L Postcentral Gyrus BA3
2	26592	38	22	-4	9E-02	2E-19	8.9	R Insula
		36	20	0	9E-02	3E-19	8.9	R Claustrum
		46	2	48	8E-02	2E-16	8.2	R Middle Frontal Gyrus BA6

		36	-2	54	8E-02	4E-14	7.5	R Precentral Gyrus BA6
		54	2	44	7E-02	3E-13	7.2	R Precentral Gyrus BA6
		48	12	26	6E-02	5E-09	5.7	R Inferior Frontal Gyrus BA9
		48	16	-2	6E-02	7E-09	5.7	R Insula BA13
		52	-10	40	5E-02	5E-07	4.9	R Precentral Gyrus BA4
3	21456	0	6	58	5E-01	0E+00	36.8	L Medial Frontal Gyrus BA6
4	14040	-28	-54	50	8E-02	1E-15	7.9	L Superior Parietal Lobule BA7
		-24	-66	52	7E-02	8E-14	7.4	L Superior Parietal Lobule BA7
		-44	-42	46	5E-02	9E-08	5.2	L Inferior Parietal Lobule BA40
		-38	-48	58	4E-02	1E-05	4.2	L Inferior Parietal Lobule BA40
		-6	-72	56	4E-02	1E-04	3.7	L Precuneus BA7
		-46	-32	58	4E-02	1E-04	3.6	L Inferior Parietal Lobule BA40
		-40	-24	58	3E-02	7E-04	3.2	L Postcentral Gyrus BA3
5	7480	44	-40	48	5E-02	3E-08	5.4	R Inferior Parietal Lobule BA40
		32	-60	54	5E-02	7E-07	4.8	R Superior Parietal Lobule BA7
		18	-68	54	4E-02	2E-06	4.6	R Precuneus BA7
		38	-50	50	4E-02	2E-06	4.6	R Inferior Parietal Lobule BA40
		26	-64	56	4E-02	2E-06	4.6	R Precuneus BA7
6	5064	-42	-62	-16	6E-02	1E-10	6.3	L Declive
		-32	-58	-30	5E-02	2E-07	5.1	L Cerebellum
		-40	-68	-28	4E-02	4E-06	4.5	L Tuber
7	3808	32	-62	-26	6E-02	8E-10	6.0	R Culmen
b. SPL L: 1325 foci, 64 experiments, 875 subjects (x=-34, y=-58, z=56)								
1	14816	-34	-58	56	3E-01	0E+00	23.4	L Superior Parietal Lobule BA7
		-42	-38	36	4E-02	7E-09	5.7	L Supramarginal Gyrus BA40
		-20	-72	52	4E-02	7E-08	5.3	L Precuneus BA7
		-50	-34	40	3E-02	3E-07	5.0	L Inferior Parietal Lobule BA40
		-42	-28	52	3E-02	7E-06	4.3	L Inferior Parietal Lobule BA40
		-42	-30	48	3E-02	9E-06	4.3	L Inferior Parietal Lobule BA40
		-46	-40	48	2E-02	2E-04	3.6	L Inferior Parietal Lobule BA40
2	8528	-2	8	48	5E-02	3E-11	6.5	L Cingulate Gyrus BA24
		4	4	60	5E-02	4E-11	6.5	R Medial Frontal Gyrus BA6
		-2	4	60	5E-02	1E-10	6.3	L Medial Frontal Gyrus BA6
		4	16	48	4E-02	2E-08	5.5	R Superior Frontal Gyrus BA6
		0	24	44	3E-02	3E-06	4.6	L Medial Frontal Gyrus BA6
3	7384	34	-54	48	5E-02	1E-11	6.7	R Superior Parietal Lobule BA7
		34	-58	56	5E-02	6E-11	6.4	R Superior Parietal Lobule BA7
		16	-64	52	3E-02	8E-06	4.3	R Precuneus BA7
		4	-64	48	2E-02	1E-04	3.7	R Precuneus BA7
4	6584	50	8	26	5E-02	9E-12	6.7	R Inferior Frontal Gyrus BA9
		44	-4	48	3E-02	1E-06	4.7	R Precentral Gyrus BA6
		36	-2	54	3E-02	2E-05	4.1	R Precentral Gyrus BA6
5	5968	-46	8	26	5E-02	1E-12	7.0	L Inferior Frontal Gyrus BA9
		-50	-8	42	3E-02	1E-05	4.2	L Precentral Gyrus BA4
		-54	2	40	3E-02	5E-05	3.9	L Precentral Gyrus BA6
		-44	24	26	2E-02	8E-05	3.8	L Middle Frontal Gyrus BA9
6	3184	-30	24	4	5E-02	2E-10	6.3	L Insula BA13
		-22	-6	6	2E-02	1E-04	3.7	L Lentiform Nucleus
		-20	2	0	2E-02	3E-04	3.4	L Lentiform Nucleus
7	2056	36	22	-10	3E-02	2E-07	5.1	R Inferior Frontal Gyrus BA47
		36	22	-2	3E-02	7E-07	4.8	R Insula
c. THA L: 2413 foci, 113 experiments, 1571 subjects (x=-14, y=-14, z=8)								
1	64248	-14	-14	8	4E-01	0E+00	30.8	L Thalamus
		12	-14	8	2E-01	0E+00	15.2	R Thalamus
		-32	20	4	1E-01	6E-22	9.6	L Insula BA13
		34	22	2	8E-02	4E-16	8.1	R Claustrum
		26	6	4	7E-02	3E-14	7.5	R Lentiform Nucleus
		50	14	-2	6E-02	3E-11	6.5	R Insula BA13
		-8	-20	-10	5E-02	2E-09	5.9	L Brainstem
		-54	12	2	5E-02	2E-08	5.5	L Precentral Gyrus BA44
		-40	2	6	5E-02	2E-08	5.5	L Insula BA13
		4	-24	-8	5E-02	5E-08	5.3	R Brainstem
		52	12	22	5E-02	7E-08	5.3	R Inferior Frontal Gyrus BA9
		54	14	8	4E-02	2E-07	5.1	R Precentral Gyrus BA44
		54	10	32	4E-02	4E-07	4.9	R Inferior Frontal Gyrus BA9
		-12	8	0	4E-02	3E-06	4.5	L Caudate

		42	2	34	3E-02	2E-04	3.6	R Precentral Gyrus BA6
2	20032	0	8	48	9E-02	1E-20	9.3	L Cingulate Gyrus BA24
		-2	0	58	8E-02	2E-16	8.1	L Medial Frontal Gyrus BA6
		8	28	28	4E-02	5E-07	4.9	R Cingulate Gyrus BA32
		20	2	56	3E-02	8E-04	3.2	R Sub-Gyral BA6
3	13944	-38	-20	58	6E-02	4E-11	6.5	L Precentral Gyrus BA4
		-30	-6	50	5E-02	9E-09	5.6	L Middle Frontal Gyrus BA6
		-44	-38	48	4E-02	8E-07	4.8	L Inferior Parietal Lobule BA40
		-50	-26	18	4E-02	2E-06	4.7	L Superior Temporal Gyrus BA41
		-56	-44	22	4E-02	1E-05	4.3	L Superior Temporal Gyrus BA13
		-38	-46	42	4E-02	2E-05	4.1	L Inferior Parietal Lobule BA40
		-56	-18	16	4E-02	2E-05	4.1	L Postcentral Gyrus BA43
		-50	-30	34	3E-02	3E-05	4.0	L Inferior Parietal Lobule BA40
		-60	-16	22	3E-02	4E-05	3.9	L Postcentral Gyrus BA43
4	3536	-52	10	32	5E-02	6E-08	5.3	L Inferior Frontal Gyrus BA9
		-46	24	26	3E-02	1E-04	3.7	L Middle Frontal Gyrus BA9
5	3136	56	-30	22	4E-02	2E-07	5.1	R Insula BA13
		64	-22	18	4E-02	5E-06	4.4	R Postcentral Gyrus BA40
6	2776	30	-58	-30	5E-02	2E-08	5.5	R Cerebellum
		22	-50	-22	4E-02	1E-05	4.2	R Culmen
		40	-52	-26	3E-02	1E-04	3.6	R Culmen
7	2192	2	-62	-18	4E-02	2E-06	4.7	R Culmen
		10	-70	-18	4E-02	6E-06	4.4	R Declive
		2	-46	-10	3E-02	7E-04	3.2	L Cerebellar Lingual
d. PreCG BA6 R: 1011 foci, 37 experiments, 569 subjects (x=54, y=-0, z=50)								
1	18304	-48	-4	52	6E-02	4E-15	7.8	L Precentral Gyrus BA4
		-34	20	4	5E-02	4E-12	6.9	L Insula BA13
		-50	8	2	4E-02	3E-09	5.8	L Insula BA13
		-54	2	38	4E-02	6E-09	5.7	L Precentral Gyrus BA6
		-56	6	6	4E-02	1E-08	5.6	L Superior Temporal Gyrus BA22
		-58	0	16	3E-02	5E-07	4.9	L Precentral Gyrus BA4
		-54	8	14	3E-02	6E-07	4.9	L Inferior Frontal Gyrus BA44
		-42	6	24	3E-02	8E-07	4.8	L Precentral Gyrus BA6
2	11216	-4	0	62	7E-02	4E-22	9.6	L Medial Frontal Gyrus BA6
		-4	18	36	4E-02	2E-08	5.5	L Cingulate Gyrus BA32
		8	12	38	3E-02	5E-08	5.3	R Cingulate Gyrus BA32
3	5928	54	0	48	2E-01	0E+00	19.6	R Precentral Gyrus BA4
		58	4	34	2E-02	3E-05	4.0	R Precentral Gyrus BA6
		34	-6	58	2E-02	4E-04	3.4	R Precentral Gyrus BA6
4	5624	50	10	2	4E-02	5E-10	6.1	R Insula BA13
		38	22	0	3E-02	9E-07	4.8	R Insula BA13
		56	14	-12	3E-02	3E-06	4.5	R Superior Temporal Gyrus BA22
5	4824	24	6	4	5E-02	5E-13	7.1	R Putamen
		14	-10	6	3E-02	1E-07	5.2	R Thalamus
		22	0	-8	2E-02	8E-05	3.8	R Globus Pallidus
6	3480	62	-32	10	3E-02	7E-08	5.3	R Superior Temporal Gyrus BA42
		56	-32	4	3E-02	9E-08	5.2	R Superior Temporal Gyrus BA22
7	3424	-52	-18	2	3E-02	2E-07	5.1	L Superior Temporal Gyrus BA22
		-62	-30	8	3E-02	1E-06	4.7	L Superior Temporal Gyrus BA42
8	2576	-22	4	6	5E-02	2E-13	7.3	L Putamen
9	1496	-52	-40	22	4E-02	2E-09	5.9	L Superior Temporal Gyrus BA13

BA, Brodmann area; ROIs, regions-of-interest; ALE, anatomic likelihood estimation; P, p-value; Z, peak z-value; R, right; L, left. **ROIs**: CRBL, cerebellum; INS, insula; MedFG, medial frontal gyrus; PreCG, precentral gyrus (primary motor cortex or M1); PUT, putamen; SPL, superior parietal lobule; STG, superior temporal gyrus (primary auditory cortex); THA, thalamus. Music-related ROIs were created in Mango (http://rii.uthscsa.edu/mango//userguide.html) with a 5mm-radius sphere.
Last search in Sleuth, 01.09.2021 (http://www.brainmap.org/sleuth/).

Supplementary Table 5. Functional characterization of brain regions resulted from primary outcomes (n = 17 ROIs) according to BrainMap database .

**1. Music perception**	
a. STG BA22 R: 824 foci, 43 experiments, 610 subjects (x=52, y=-20, z=4)	
Action	Execution, speech, motor learning, observation
Cognition	Attention, phonology, semantics, speech, explicit memory, working memory, **music**, reasoning, spatial
Emotion	Anxiety, sadness, positive emotion, reward/gain, valence
Interoception	Sexuality
Perception	Audition, somesthesis, vision, shape
Paradigms	Acupuncture, classical conditioning, cued explicit recognition/recall, drawing, emotion induction, encoding, face discrimination, finger tapping/button pressing, flexion/extension, mental rotation, **music comprehension, music production**, n-back, naming (overt), oddball discrimination, paired associate recall, passive listening, passive viewing, phonological discrimination, pitch discrimination, reading overt, reading covert, reasoning/problem solving, recitation/repetition, reward, semantic discrimination, sequence recall/learning, sexual arousal/gratification, tone discrimination, visuospatial attention
b. STG BA22 L: 1143 foci, 60 experiments, 850 subjects (x=-54, y=-16, z=2)	
Action	Execution, speech, inhibition, observation
Cognition	Attention, phonology, semantics, speech, explicit memory, working memory, **music**, reasoning, somatic, spatial
Emotion	Anger, fear, guilt, sadness, positive emotion, happiness, reward/gain, valence
Interoception	Sexuality
Perception	Audition, vision, shape
Paradigms	Counting/calculation, cued explicit recognition/recall, emotion induction, encoding, face discrimination, film viewing, finger tapping/button pressing, flexion/extension, go/no-go, hand-eye coordination, imagined objects/scene, mental rotation, **music comprehension, music production**, oddball discrimination, paired associate recall, passive listening, passive viewing, phonological discrimination, pitch discrimination, reading overt, reading covert, reasoning/problem solving, recitation/repetition, reward, semantic discrimination, sequence recall/learning, sexual arousal/gratification, tone discrimination, visuospatial attention, word generation (covert), word generation (overt)
c. MedFG BA6 L: 1773 foci, 58 experiments, 838 subjects (x=-2, y=-2, z=66)	
Action	Execution, speech, imagination, inhibition, motor learning, observation
Cognition	Attention, language, phonology, semantics, speech, explicit memory, working memory, **music**, reasoning, social cognition, somatic, temporal
Emotion	Sadness, positive emotion
Interoception	-
Perception	Audition, gustation, somesthesis, pain, vision, color, motion
Paradigms	Acupuncture, affective words, counting/calculation, cued explicit recognition/recall, deception, delayed match to sample, emotion induction, episodic recall, estimation, face discrimination, finger tapping/button pressing, flanker, flexion/extension, go/no-go, imagined movement, imagined objects/scene, tongue movement, **music comprehension, music production**, n-back, pain discrimination, passive listening, passive viewing, pitch discrimination, manual tracking, reading overt, reading covert, recitation/repetition, saccades, semantic discrimination, sequence recall/learning, tactile discrimination, tone discrimination, visual object identification, visual tracking, visuospatial attention, writing
d. PUT R: 1821 foci, 70 experiments, 1052 subjects (x=22, y=8, z=6)	
Action	Execution, speech, imagination, inhibition, observation
Cognition	Attention, orthography, phonology, semantics, speech, explicit memory, working memory, **music**, reasoning, somatic, spatial, temporal
Emotion	Anger, fear, reward/gain, valence
Interoception	Baroregulation, respiration regulation, sexuality
Perception	Audition, gustation, olfaction, somesthesis, pain, vision, color, motion, shape
Paradigms	Affective pictures, affective words, anti-saccades, chewing/swallowing, counting/calculation, cued explicit recall, encoding, face discrimination, film viewing, finger tapping/button press, flexion/extension, gambling, go/no-go, hypercapnia, imagined movement, isometric force, magnitude comparison, **music comprehension, music production**, olfactory discrimination, pain discrimination, paired associate recall, passive listening, passive viewing, pitch discrimination, pointing, reading covert, reading overt, reasoning, recitation, reward, saccades, semantic discrimination, sequence recall/learning, sexual arousal, Stroop-color, tactile discrimination, task switching, tone discrimination, visual object identification, visuospatial attention, word generation
e. PUT L: 1493 foci, 69 experiments, 1139 subjects (x=-22, y=4, z=6)	
Action	Execution, speech, inhibition, preparation
Cognition	Attention, phonology, semantics, speech, explicit memory, working memory, **music**, reasoning, spatial, temporal
Emotion	Negative emotion, sadness, positive emotion, happiness, reward/gain, valence
Interoception	Gastrointestinal/Genitourinary, hunger, sexuality
Perception	Audition, gustation, olfaction, somesthesis, pain, vision, color, motion, shape
Paradigms	Acupuncture, affective pictures, chewing/swallowing, classical conditioning, cued explicit recognition/recall, deception, delayed match to sample, emotion induction, encoding, episodic recall, estimation, face discrimination, film viewing, finger

	tapping/button pressing, flanker, flexion/extension, go/no-go, hunger, imagined movement, isometric force, micturition, **music comprehension**, n-back, naming covert, olfactory discrimination, orthographic discrimination, pain discrimination, passive listening, passive viewing, phonological discrimination, pitch discrimination, reading overt, recitation/repetition, reward, semantic discrimination, sexual arousal/gratification, Stroop-color, task switching, taste, tone discrimination, visual object identification, visuospatial attention, Wisconsin card sorting, word generation (covert)
f. CRBL L: 1201 foci, 47 experiments, 688 subjects (x=-28, y=-64, z=-26)	
Action	Execution, speech, imagination, inhibition, preparation
Cognition	Attention, language orthography, phonology, semantics, speech, explicit memory, working memory, **music**, reasoning
Emotion	Negative emotion, anger, sadness, positive emotion, happiness, valence
Interoception	Gastrointestinal/Genitourinary, sexuality, sleep, vestibular
Perception	Audition, somesthesis, pain, vision, shape
Paradigms	Affective words, counting/calculation, cued explicit recognition/recall, deception, delayed match to sample, divided auditory attention, emotion induction, encoding, episodic recall, face discrimination, film viewing, finger tapping/button pressing, go/no-go, imagined movement, micturition, **music comprehension, music production**, pain discrimination, paired associate recall, passive listening, passive viewing, pitch discrimination, reading overt, reading covert, recitation/repetition, reward, semantic discrimination, sequence recall/learning, sexual arousal/gratification, tactile discrimination, tone discrimination, vestibular stimulation, visual object identification, visuospatial attention, word generation (covert)
g. INS L: 1787 foci, 87 experiments, 1332 subjects (x=-32, y=18, z=10)	
Action	Execution, speech, imagination, inhibition, observation, preparation
Cognition	Attention, orthography, phonology, semantics, speech, syntax, explicit memory, implicit memory, working memory, **music**, reasoning, social cognition, somatic, spatial
Emotion	Negative emotion, anxiety, embarrassment, fear, positive emotion, happiness, reward/gain, valence
Interoception	Sexuality
Perception	Audition, gustation, olfaction, pain, vision, color, motion, shape
Paradigms	Affective words, chewing/swallowing, classical conditioning, counting/calculation, cued explicit recognition/recall, deception, delayed match to sample, driving, emotion induction, encoding, face discrimination, figurative language, film viewing, finger tapping/button pressing, flanker, gambling, go/no-go, grasping, imagined movement, imagined objects/scene, isometric force, multi-tasking, **music comprehension**, oddball discrimination, olfactory discrimination, orthographic discrimination, pain discrimination, passive listening, passive viewing, phonological discrimination, reading overt, reading covert, reasoning/problem solving, reward, saccades, semantic discrimination, sexual arousal/gratification, Stroop-color, task-switching, taste, theory of mind, tone discrimination, visual object identification, visuospatial attention, Wisconsin card sorting test, word generation (overt)
h. PreCG R: 1613 foci, 63 experiments, 931 subjects (x=54, y=0, z=46)	
Action	Execution, speech, imagination, inhibition, motor learning, observation, preparation
Cognition	Attention, orthography, phonology, semantics, speech, explicit memory, implicit memory, working memory, **music**, reasoning, social cognition, temporal
Emotion	Intensity, negative emotion, embarrassment, fear, sadness, positive emotion, happiness, reward/gain
Interoception	-
Perception	Audition, somesthesis, pain, vision, motion
Paradigms	Affective pictures, affective words, chewing/swallowing, counting/calculation, cued explicit recognition/recall, deception, delay discounting, delayed match to sample, driving, emotion induction, encoding, episodic recall, face discrimination, film viewing, finger tapping/button pressing, flexion/extension, go/no-go, grasping, imagined movement, multitasking, **music comprehension, music production**, pain discrimination, passive listening, passive viewing, pitch discrimination, pointing, reading overt, reading covert, reasoning/problem solving, recitation/repetition, reward, saccades, semantic discrimination, Stroop-color, theory of mind, tone discrimination, visual pursuit, visuospatial attention, word stem generation
**2. Music production**	
a. STG BA42 R: 753 foci, 32 experiments, 455 subjects (x=66, y=-24, z=10)	
Action	Execution, speech, observation
Cognition	Attention, language, phonology, semantics, speech, syntax, explicit memory, working memory, **music**, reasoning, social cognition
Emotion	Negative emotion, anxiety, disgust, sadness, reward/gain
Interoception	Thermoregulation
Perception	Audition, pain, vision, motion, shape
Paradigms	Affective pictures, counting/calculation, cued explicit recognition/recall, emotion induction, face discrimination, film viewing, finger tapping/button pressing, go/no-go, **music comprehension, music production**, naming, oddball discrimination, pain discrimination, passive listening, passive viewing, phonological discrimination, pitch discrimination, reading overt, reasoning/problem solving, recitation/repetition, reward, semantic discrimination, sequence recall, theory of mind, tone discrimination, visual object identification, visual pursuit/tracking, word generation (overt)
b. PreCG L: 1372 foci, 79 experiments, 1034 subjects (x=-48, y=0, z=42)	
Action	Execution, speech, imagination, inhibition, observation
Cognition	Attention, language, orthography, phonology, semantics, speech, syntax, explicit memory, working memory, **music**, reasoning, temporal

Emotion	Fear, sadness
Interoception	Respiration regulation, sexuality
Perception	Audition, somesthesis, vision, motion, shape
Paradigms	Counting/calculation, cued explicit recognition/recall, delayed match to sample, encoding, face discrimination, film viewing, finger tapping/button pressing, go/no-go, hypercapnia/air hunger, imagined movement, mental rotation, **music comprehension, music production**, n-back, naming covert, naming overt, orthographic discrimination, paired associate recall, passive viewing, phonological discrimination, pitch discrimination, pointing, pursuit rotor/manual tracking, reading overt, reading covert, reasoning/problem solving, recitation/repetition, saccades, semantic discrimination, sequence recall/learning, sexual arousal/gratification, tactile discrimination, task-switching, visual pursuit/tracking, visuospatial attention, word generation (covert), word generation (overt)
c. MedFG BA6 L: 403 foci, 21 experiments, 261 subjects (x=-10, y=-10, z=52)	
Action	Execution, imagination, inhibition
Cognition	Attention, orthography, semantics, explicit memory, working memory, **music**, social cognition
Emotion	Positive emotion, reward/gain
Interoception	Respiration regulation
Perception	Somesthesis, vision, color
Paradigms	Affective pictures, affective words, competition/cooperation, deception, finger tapping/button pressing, flexion/extension, go/no-go, hypercapnia/air hunger, imagined movement, isometric force, **music comprehension, music production**, n-back, reading covert, reward, semantic discrimination, sequence recall/learning, tactile discrimination, trauma recall, videogames, visual pursuit/tracking, visuospatial attention
d. STG BA41 L: 529 foci, 38 experiments, 522 subjects (x=-42, y=-28, z=6)	
Action	Execution, speech, imagination, observation, preparation
Cognition	Attention, language, phonology, semantics, speech, explicit memory, working memory, **music**, social cognition, spatial
Emotion	Negative emotion, anger, anxiety, fear, sadness, positive emotion, happiness
Interoception	-
Perception	Audition, somesthesis, vision
Paradigms	Affective words, deception, emotion induction, episodic recall, face discrimination, film viewing, finger tapping/button pressing, fixation, flexion/extension, hand-eye coordination, imagined movement, imagined objects/scenes, **music comprehension, music production**, naming overt, oddball discrimination, pain discrimination, passive listening, passive viewing, phonological discrimination, pitch discrimination, recitation/repetition, semantic discrimination, tone discrimination, visual object identification, visuospatial attention, word generation covert
e. PreCG R: 1105 foci, 51 experiments, 846 subjects (x=54, y=4, z=32)	
Action	Execution, speech, imagination, inhibition, observation
Cognition	Attention, orthography, phonology, speech, syntax, explicit memory, working memory, **music**, reasoning, social cognition, temporal
Emotion	Negative emotion, anger, fear, sadness, positive emotion, happiness, valence
Interoception	Respiration regulation, sexuality
Perception	Audition, gustation, pain, vision, motion
Paradigms	Affective pictures, affective words, chewing/swallowing, counting/calculation, cued explicit recognition/recall, delayed match to sample, emotion induction, encoding, face discrimination, film viewing, finger tapping/button pressing, fixation, flexion/extension, go/no-go, grasping, hypercapnia/air hunger, imagined movement, imagined objects/scenes, **music comprehension, music production**, orthographic discrimination, pain discrimination, paired passive viewing, phonological discrimination, pitch discrimination, pointing, pursuit rotor/manual tracking, reading overt, reading covert, reasoning/problem solving, recitation/repetition, saccades, sexual arousal, theory of mind, visual object identification, visual pursuit/tracking, visuospatial attention, word generation (overt), word completion covert, word completion overt, writing
**3. Music imagery**	
a. MedFG L: 3287 foci, 161 experiments, 2114 subjects (x=0, y=6, z=58)	
Action	Execution, speech, imagination, inhibition, observation, preparation
Cognition	Attention, language, phonology, semantics, speech, syntax, explicit memory, working memory, **music**, reasoning, social cognition, somatic, spatial, temporal
Emotion	Negative emotion, anger, anxiety, sadness, happiness, humor, reward/gain, valence
Interoception	Heartbeat detection, sexuality
Perception	Audition, somesthesis, pain, vision, motion, shape
Paradigms	Affective pictures, anti-saccades, chewing/swallowing, classical conditioning, competition/cooperation, counting/calculation, cued explicit recognition/recall, delayed match to sample, drawing, emotion induction, encoding, face discrimination, film viewing, finger tapping/button pressing, fixation, flexion/extension, go/no-go, grasping, imagined movement, imagined objects/scenes, isometric force, lexical decision, mental rotation, **music comprehension, music production,** n-back,, naming covert, naming overt, orthographic discrimination, pain discrimination, paired associate recall, passive listening, phonological discrimination, pitch discrimination, pointing, reading overt, reading covert, reasoning/problem solving, recitation/repetition, reward, saccades, semantic discrimination, sequence recall/learning, sexual arousal/gratification, Stroop-color, Stroop-emotional, syntactic discrimination, tactile discrimination, task-switching, tone discrimination, Tower of London, videogames, visual object identification, visuospatial attention, Wisconsin card sorting test, word generation (covert), word generation (overt), word completion

b. SPL L: 1325 foci, 64 experiments, 875 subjects (x=-34, y=-58, z=56)	
Action	Execution, speech, imagination, inhibition, observation
Cognition	Attention, orthography, phonology, semantics, speech, explicit memory, working memory, **music**, reasoning, social cognition, somatic, spatial
Emotion	Negative emotion, anxiety, disgust, positive emotion, happiness, reward/gain
Interoception	Sexuality
Perception	Audition, gustation, somesthesis, pain, vision, color, motion, shape
Paradigms	Affective pictures, anti-saccades, counting/calculation, cued explicit recognition/recall, delay discounting, delayed match to sample, emotion induction, encoding, face discrimination, film viewing, finger tapping/button pressing, flexion/extension, gambling, go/no-go, imagined movement, isometric force, magnitude comparison, mental rotation, **music comprehension**, n-back, naming, orthographic discrimination, pain discrimination, paired associate recall, passive viewing, pitch discrimination, reading overt, reading covert, reasoning/problem solving, reward, saccades, semantic discrimination, sequence recall/learning, sexual arousal/gratification, Stroop-color, tactile discrimination, task-switching, visual object identification, visual pursuit/tracing, visuospatial attention, word generation (overt)
c. THA L: 2413 foci, 113 experiments, 1571 subjects (x=-14, y=-14, z=8)	
Action	Execution, speech, imagination, inhibition, motor learning
Cognition	Attention, language, orthography, phonology, semantics, speech, explicit memory, working memory, **music**, reasoning, social cognition, temporal
Emotion	Negative emotion, anxiety, fear, sadness, positive emotion, happiness, reward/gain, valence
Interoception	Gastrointestinal/Genitourinary, respiration regulation, sleep, thermoregulation
Perception	Audition, gustation, somesthesis, pain, vision, color, motion, shape
Paradigms	Affective words, anti-saccades, classical conditioning, counting/calculation, cued explicit recognition/recall, delay discounting, delayed match to sample, emotion induction, encoding, episodic recall, face discrimination, film viewing, finger tapping/button pressing, flanker, flexion/extension, gambling, go/no-go, grasping, hypercapnia, air hunger, imagined objects/scene, isometric force, lexical decision, micturition, motor learning, **music comprehension, music production**, n-back, naming, oddball discrimination, orthographic discrimination, pain discrimination, paired associate recall, passive listening, passive viewing, phonological discrimination, pointing, pursuit rotor/manual tracking, reading overt, reading covert, reasoning/problem solving, recitation/repetition, reward, saccades, sequence recall/learning, Stroop-counting, tactile discrimination, task-switching, taste, tone discrimination, transcranial magnetic stimulation, trauma recall, videogames, visual object identification, visual pursuit/tracking, visuospatial attention, word generation (covert), word generation (overt), writing
d. PreCG BA6: 1011 foci, 37 experiments, 569 subjects (x=54, y=-0, z=50)	
Action	Execution, speech, imagination, motor learning, observation
Cognition	Attention, orthography, semantics, speech, explicit memory, working memory, **music**, reasoning
Emotion	Intensity, negative emotion, sadness, positive emotion, happiness, reward/gain
Interoception	Thermoregulation
Perception	Audition, olfaction, pain, vision, motion
Paradigms	Affective pictures, affective words, chewing/swallowing, counting/calculation, cued explicit recognition/recall, delay discounting, delayed match to sample, emotion induction, face discrimination, film viewing, finger tapping/button pressing, flexion/extension, go/no-go, imagined movement, multi-tasking, **music comprehension, music production**, olfactory discrimination, pain discrimination, passive listening, passive viewing, phonological discrimination, pitch discrimination, reading overt, reading covert, reasoning/problem solving, recitation/repetition, reward, saccades, semantic discrimination, tactile discrimination, task-switching, tone discrimination, visuospatial attention

BA, Brodmann area; ROIs, regions-of-interest; ALE, anatomic likelihood estimation; P, p-value; Z, peak z-value; R, right; L, left. **ROIs**: CRBL, cerebellum; INS, insula; MedFG, medial frontal gyrus; PreCG, precentral gyrus (primary motor cortex or M1); PUT, putamen; SPL, superior parietal lobule; STG, superior temporal gyrus (primary auditory cortex); THA, thalamus. Music-related ROIs were created in Mango (http://rii.uthscsa.edu/mango//userguide.html) with a 5mm-radius sphere.

Last search in Sleuth, 01.09.2021 (http://www.brainmap.org/sleuth/).

Supplementary Table 6. FSN robustness assessment for significant ALE maps of music perception, production, and imagery

Cluster number	Volume (mm^3)	MNI coordinates x	y	z	ALE	Label (Side, region)	Contributing studies (k)	FSN
1. Music perception: 1898 foci, 105 experiments, 2035 subjects, minimum FSN = 32								
1	24296	52	-20	4	1E-01	R Superior Temporal Gyrus BA13	75	292
2	23864	-54	-16	2	1E-01	L Superior Temporal Gyrus BA22	79	292
3	6136	-2	-2	66	5E-02	L Medial Frontal Gyrus BA6	31	105
4	3328	22	8	6	6E-02	R Putamen	21	105
5	2304	-22	4	6	5E-02	L Putamen	16	50
6	1920	-28	-64	-26	5E-02	L Cerebellum	13	50
7	1816	-32	18	10	4E-02	L Insula BA13	11	75
8	1728	54	0	46	6E-02	R Precentral Gyrus BA4	14	35
2. Music production: 499 foci, 19 experiments, 292 subjects, minimum FSN = 6								
1	3512	66	-24	10	2E-02	R Superior Temporal Gyrus BA42	8	75
2	2920	-48	0	42	2E-02	L Precentral Gyrus BA6	7	57
3	2080	-10	-10	52	2E-02	L Medial Frontal Gyrus BA6	9	61
4	1912	-42	-28	6	3E-02	L Superior Temporal Gyrus BA13	5	40
5	1072	54	4	32	2E-02	R Precentral Gyrus BA6	5	<6
3. Music imagery: 263 foci, 15 experiments, 189 subjects, minimum FSN = 5								
1	3256	0	6	58	2E-02	L Medial Frontal Gyrus BA6	10	56
2	3128	-34	-58	56	2E-02	L Superior Parietal Lobule BA7	9	30
3	1384	-14	-14	8	2E-02	L Thalamus	3	35
4	944	54	0	50	2E-02	L Precentral Gyrus BA6	6	10

FSN, Fail-Safe N analysis; ALE, anatomic likelihood estimation; BA, Brodmann area; R, right; L, left.

Citations of included studies

Abbreviations

ACC	anterior cingulate cortex
AF	arcuate fasciculus
AnG	angular gyrus
CalC	calcarine cortex
CC	corpus callosum
CAU	caudate
CLAU	claustrum
CRBL	cerebellum
CST	corticospinal tract
CUN	cuneus
DLPFC	dorsolateral prefrontal cortex
EC	entorhinal cortex
Fmaj	forceps major
Fmin	forceps minor
FO	frontal operculum
FusG	fusiform gyrus
GP	globus pallidus
HG	Helsch's gyrus
HIPP	hippocampus
IC	internal capsule
IF	inferior colliculus
IFG	inferior frontal gyrus
IFOF	inferior fronto-occipital fasciculus
ILF	inferior longitudinal fasciculus
INS	insula
IPL	inferior parieal lobule
ITG	inferior temporal gyrus
LG	lingual gyrus
LOC	lateral occipital cortex
MB	midbrain
MCC	middle cingulate cortex
MCP	middle cerebellar peduncle
MedFG	medial frontal gyrus
MidFG	middle frontal gyrus
MidTG	middle temporal gyrus
OFC	orbitofrontal cortex
PaHIPP	parahippocampal gyrus
PCC	posterior cingulate cortex
PMC	premotor cortex
PO	parietal operculum
PostCG	postcentral gyrus (primary somatosensory cortex or SI)
PP	planum polare
PreCG	precentral gyrus (primiary motor cortex or M1)
PT	planum temporale
PUT	putamen
RN	red nucleus
SCP	superior cerebellar peduncle
SFG	superior frontal gyrus
SII	secondary somatosensory cortex
SLF	superior longitudial fasciculus
SMA	supplementary motor area
SMG	supramarginal gyrus
SPL	superior parietal lobule
STG	superior temporal gyrus
STS	superior temporal sulcus
THA	thalamus
TP	temporal pole
TPG	temporoparietal junction
VER	Vermis

References

1. Poldrack, R. A. *et al.* Guidelines for reporting an fMRI study. *Neuroimage* **40**, 409–414 (2008).
2. Nichols, T. E. *et al.* Best practices in data analysis and sharing in neuroimaging using MRI. *Nat. Neurosci.* **20**, 299–303 (2017).
3. Laird, A. R. *et al. User Manual for Sleuth 2.0*.
4. Eickhoff, S. B., Bzdok, D., Laird, A. R., Kurth, F. & Fox, P. T. Activation likelihood estimation meta-analysis revisited. *Neuroimage* **59**, 2349–2361 (2012).
5. Laird, A. R. *et al.* Networks of task co-activations. *Neuroimage* **80**, 505–514 (2013).
